# Determining the Number of Latent Factors in Statistical Multi-Relational Learning

**Published:** 2019

**Authors:** Chengchun Shi, Wenbin Lu, Rui Song

**Affiliations:** Department of Statistics, North Carolina State University, Raleigh, NC 27695, USA

**Keywords:** Information criteria, Knowledge graph, Model selection consistency, RESCAL model, Statistical relational learning, Tensor factorization

## Abstract

Statistical relational learning is primarily concerned with learning and inferring relationships between entities in large-scale knowledge graphs. Nickel et al. (2011) proposed a RESCAL tensor factorization model for statistical relational learning, which achieves better or at least comparable results on common benchmark data sets when compared to other state-of-the-art methods. Given a positive integer *s*, RESCAL computes an *s*-dimensional latent vector for each entity. The latent factors can be further used for solving relational learning tasks, such as collective classification, collective entity resolution and link-based clustering.

The focus of this paper is to determine the number of latent factors in the RESCAL model. Due to the structure of the RESCAL model, its log-likelihood function is not concave. As a result, the corresponding maximum likelihood estimators (MLEs) may not be consistent. Nonetheless, we design a specific pseudometric, prove the consistency of the MLEs under this pseudometric and establish its rate of convergence. Based on these results, we propose a general class of information criteria and prove their model selection consistencies when the number of relations is either bounded or diverges at a proper rate of the number of entities. Simulations and real data examples show that our proposed information criteria have good finite sample properties.

## 1. Introduction

Relational data is becoming ubiquitous in artificial intelligence and social network analysis. These data sets are in the form of graphs, with nodes and edges representing entities and relationships, respectively. Recently, a number of companies have developed and released their knowledge graphs, including the Google Knowledge Graph, Microsoft Bing’s Satori Knowledge Base, Yandex’s Object Answer, the Linkedln Knowledge Graph, etc. These knowledge graphs are graph-structured knowledge bases that store factual information as relationships between entities. They are created via the automatic extraction of semantic relationships from semi-structured or unstructured text (see Section II.C in Nickel et al., 2016). The data may be incomplete, noisy and contain false information. It is therefore of great importance to infer the existence of a particular relationship to improve the quality of these extracted information.

Statistical relational learning is primarily concerned with learning from relational data sets, and solving tasks such as predicting whether two entities are related (link prediction), identifying equivalent entities (entity resolution), and grouping similar entities based on their relationships (link-based clustering). Statistical relational models can be roughly divided into three categories: the relational graphical models, the latent class models and the tensor factorization models. Relational graphical models include probabilistic relational models (Getoor and Mihalkova, 2011) and Markov logic networks (MLN, Richardson and Domingos, 2006). These models are constructed via Bayesian or Markov networks. In latent class models, each entity is assigned to one of the latent classes and the probability of a relationship between entities depends on their corresponding classes. Two important examples include the stochastic block model (SBM, Nowicki and Snijders, 2001) and the infinite relational model (IRM, Kemp et al., 2006). IRM can be viewed as a nonparametric extension of SBM where the total number of clusters is not prespecified. Both models have received considerable attentions in the statistics and machine learning literature for community detection in networks.

Tensors are multidimensional arrays. Tensor factorization methods such as CANDE-COMP/PARAFAC (CP, Harshman and Lundy, 1994), Tucker (Tucker, 1966) and their extensions have found applications in a variety of fields. Kolda and Bader (2009) presented a thorough overview of tensor decompositions and their applications. Recently, tensor factorizations are being actively studied in the statistics literature and have becoming an emerging field of statistics. To name a few, Chi and Kolda (2012) developed a Poisson tensor factorization model for sparse count data. Yang and Dunson (2016) proposed a conditional tensor factorization model for high-dimensional classification with categorical predictors. Sun et al. (2017) proposed a sparse tensor decomposition method by incorporating a truncation step into the tensor power iteration step.

Relational data sets are typically expressed as (subject, predicate, object) triples and can be grouped as a third-order tensor. As a result, tensor factorization methods can be naturally applied to these data sets. Nickel (2013) proposed a RESCAL factorization model for statistical relational learning. Compared to other tensor factorization approaches such as CP and Tucker methods, RESCAL is more capable of detecting the correlations produced between multiple interconnected nodes. For relational data consisting of *n* entities, *K* types of relations, and a positive integer *s*, RESCAL computes an *n* × *s* factor matrix and an *s × s × K* core tensor. The factor matrix and the core tensor can be further used for link prediction, entity resolution and link-based clustering. Nickel et al. (2011) showed that a linear RESCAL model achieved better or comparable results on common benchmark data sets when compared to other existing methods such as MLN, DEDICOM (Harshman, 1978), IRM, CP, MRC (Kok and Domingos, 2007), etc. It was shown in Nickel and Tresp (2013) that a logistic RESCAL model could further improve the link prediction results.

Central to the empirical validity of RESCAL is the correct specification of the number of latent factors. Nickel et al. (2011) proposed to select this parameter via cross-validation. As commonly known for cross-validation methods, there’s no theoretical guarantee against overestimation. Besides, cross-validation can be computationally expensive, especially for large *n* and *K*. In the literature, model selection is less studied for tensor factorization methods. Allen (2012) and Sun et al. (2017) proposed to use Bayesian information criteria (BIC, Schwarz, 1978) for sparse CP decomposition. However, no theoretical results were provided for BIC. Indeed, we show in this paper that a BIC-type criterion may fail for the RESCAL model.

The contribution of this paper is twofold. First, we propose a general class of information criteria for the RESCAL model and prove their model selection consistency. Although we focus on the RESCAL model, our information criteria can be extended to select models for general tensor factorization methods with slight modification. The problem is nonstandard and challenging since both the factor matrix and the core tensor are not observed and need to be estimated. Besides, the model parameters are non-identifiable. Moreover, the derivation of model/tuning parameter selection consistency of information criteria usually relies on the (uniform) consistency of estimated parameters. For example, Fan and Tang (2013) derived the uniform consistency of the maximum likelihood estimators (MLEs) to prove the consistency of GIC (see Proposition 2 in that paper). Zhang et al. (2016) established the uniform consistency of the support vector machine solutions to prove the consistency of SVMIC_*h*_ (see Lemma 2 in that paper). The consistency of these estimators are due to the concavity (convexity) of the likelihood (or the empirical loss) functions. In contrast, for most tensor decomposition models including RESCAL, the likelihood (or the empirical loss) function is usually non-concave (non-convex) and may have multiple local solutions. As a result, the corresponding global maximizer (minimizer) may not be consistent even with the identifiability constraints. It remains unknown how to establish the consistency of the information criterion without consistency of the estimator. A key innovation in our analysis is to design a “proper” pseudometric and show that the global optimum is consistent under this specific pseudometric. We further establish the rate of convergence of the global optimum under this pseudometric as a function of *n* and *K*. Based on these results, we establish the consistency of our information criteria when *K* is either bounded or diverges at a proper rate of *n*. No parametric assumptions are imposed on the latent factors. Second, we introduce a scalable algorithm for estimating the parameters in the logistic RESCAL model. Despite the fact that a linear RESCAL model can be conveniently solved by an alternating least square algorithm (Nickel et al., 2011), there are lack of optimization algorithms for solving general RESCAL models. The proposed algorithm is based on the alternating direction method of multipliers (ADMM, Boyd et al., 2011) and can be implemented in a parallelized fashion.

The rest of the paper is organized as follows. We formally introduce the RESCAL model and study the parameter identifiability in Section 2. Our information criteria are presented in Section 3 and their model selection properties are investigated. Numerical examples are presented in Section 4 to examine the finite sample performance of the proposed information criteria. Section 5 concludes with a summary and discussion of future extensions. All the proofs are given in the Appendix.

## 2. The RESCAL Model

This section is structured as follows. We introduce the RESCAL model in Section 2.1. In Section 2.2, we study the identifiability of parameters in the model.

### 2.1. Model Setup

In knowledge graphs, facts can be expressed in the form of (subject, predicate, object) triples, where subject and object are entities and predicate is the relation between entities. For example, consider the following sentence from Wikipedia:

Jon Snow is a fictional character in the A Song of Ice and Fire series of fantasy novels by American author George R. R. Martin, and its television adaptation Game of Thrones.

The information contained in this message can be summarized into the following set of (subject, predicate, object) triples:

**Table:** 

Subject	Predicate	Object
Jon Snow	character in	A Song of Ice and Fire
Jon Snow	character in	Game of Thrones
A Song of Ice and Fire	genre	novel
Game of Thrones	genre	television series
George R.R. Martin	author of	A Song of Ice and Fire
George R.R. Martin	profession	novelist

In this example, we have a total of 7 entities, 4 types of relations and 6 triples. More generally, let $E=\left\{e_{1},\ldots ,e_{n}\right\}$ denote the set of all entities and $R=\left\{r_{1},\ldots ,r_{K}\right\}$ denote the set of all relation types. The number of relations *K* is either bounded or diverges with *n*. Assuming non-existing triples indicate false relationships, we can construct a third-order binary tensor

$$Y={\left\{Y_{ijk}\right\}}_{i,j\in \{1,\ldots ,n\},k\in \{1,\ldots ,K\}},$$

such that

$$Y_{ijk}=\{\begin{array}{ll} 1, & \text{if a triple}\;\left(e_{i},r_{k},e_{j}\right)\;\text{exists,}\\ 0, & \text{otherwise}. \end{array}$$

The RESCAL model is defined as follows. For each entity *e*_*i*_, a latent vector $a_{i,0}\in {\mathbb{R}}^{s_{0}}$ is generated. The *Y*_*ijk*_’s are assumed to be conditionally independent given all latent factors ${\left\{a_{i,0}\right\}}_{i=1}^{n}$. Besides, it is assumed that

(1) $$\mathrm{Pr}\left(Y_{ijk}=1|{\left\{a_{i,0}\right\}}_{i=1}^{n}\right)=g(a_{i,0}^{T}R_{k,0}a_{j,0}),$$

for some strictly monotone link function g and *s*_0_ × *s*_0_ matrices $R_{1,0},\ldots ,R_{K,0}$. In the above model, ***a***_*i*,0_ corresponds to the latent representation of the *i*th entity and ***R***_*k*,0_ specifies how these ***a***_*i*,0_’s interact for the *k*-th relation. To account for asymmetric relations, we do not restrict ***R***_*k*,0_’s to symmetric matrices. When the relations are symmetric, i.e.,

$$\mathrm{Pr}\left(Y_{ijk}=1|{\left\{a_{i,0}\right\}}_{i=1}^{n}\right)=\mathrm{Pr}\left(Y_{jik}=1|{\left\{a_{i,0}\right\}}_{i=1}^{n}\right),\;\forall i,j,k,$$

one can impose the symmetry constraints and obtain a similar derivation.

For continuous *Y*_*ijk*_, a related tensor factorization model is the TUCKER-2 decomposition, which decomposes the tensor into

(2) $$Y_{ijk}=a_{i,0}^{T}R_{k,0}b_{j,0}+e_{ijk},\;\forall i,j,k,$$

for some $a_{1,0},\ldots ,a_{n,0}\in {\mathbb{R}}^{s_{1}},\;b_{1,0},\ldots ,b_{n,0}\in {\mathbb{R}}^{s_{2}},\;R_{1,0},\ldots ,R_{K,0}\in {\mathbb{R}}^{s_{1}\times s_{2}}$ and some (random) errors ${\left\{e_{ijk}\right\}}_{ijk}$. By Equation 1, RESCAL can be interpreted as a “nonlinear” TUCKER-2 model with the additional constraints that *s*_1_ = *s*_2_ = *s*_0_ and $a_{i,0}=b_{i,0},\;\forall i$.

CP decomposition is another important tensor factorization method that decomposes a tensor into a sum of rank-1 tensors. It assumes that

$$Y_{ijk}=\sum_{s=1}^{s_{0}}a_{i,s}b_{j,s}r_{k,s}+e_{ijk},$$

for some ${\left\{a_{i,s}\right\}}_{i,s},{\left\{b_{j,s}\right\}}_{j,s},{\left\{r_{k,s}\right\}}_{k,s}$ and ${\left\{e_{ijk}\right\}}_{ijk}$. Define $a_{i,0}={\left(a_{i,1},\ldots ,a_{i,s_{0}}\right)}^{T}$ and $b_{i,0}={\left(b_{i,1},\ldots ,b_{i,s_{0}}\right)}^{T}$. In view of Equation 2, CP is a special TUCKER-2 model with the constraints that *s*_1_ = *s*_2_ = *s*_0_ and $R_{k,0}=\mathrm{diag}\left(r_{k,1},\ldots ,r_{k,s_{0}}\right)$ where $\text{diag}\left(r_{k,1},\ldots ,r_{k,s_{0}}\right)$ is a diagonal matrix with the *s*th diagonal elements being *r*_*k,s*_.

In this paper, the proposed information criteria are designed in particular for the RESCAL model. However, they can be extended to estimate *s*_0_ in a more general tensor factorization framework including CP and TUCKER-2 models. We discuss this further in Section 5.

### 2.2. Identifiability

The parameterization in Equation 1 is not identifiable. To see this, for any nonsingular matrix $C\in {\mathbb{R}}^{s_{0}\times s_{0}}$, we define $a_{i}=C^{-1}a_{i,0},R_{k}=C^{T}R_{k,0}C,\;\forall i,k$. Observe that

$$a_{i,0}^{T}R_{k,0}a_{j,0}=a_{i}^{T}R_{k}a_{j},\;\forall i,j,k$$

and hence we have

$$\mathrm{Pr}\left(Y_{ijk}=1\right)=\text{g}\left(a_{i}^{T}R_{k}a_{j}\right).$$

Let $A_{0}={\left[a_{1,0},\ldots ,a_{n,0}\right]}^{T}$. We impose the following condition.

(A0) (i) Assume ***A***_0_ has full column rank. (ii) Assume the matrix $\left[R_{1,0}^{T},\ldots ,R_{K,0}^{T}\right]$ has full row rank.

(A0)(i) requires the latent factors to be linearly independent. (A0)(ii) holds when at least one of the ***R***_*k*,0_’s has full rank. Under Condition (A0), the following lemma states that the RESCAL model is identifiable up to a nonsingular linear transformation. In Section B.1 of the Appendix, we show (A0) is also necessary to guarantee such identifiability when $R_{1,0},\ldots ,R_{K,0}$ are symmetric.

#### Lemma 1 (Identifiability).

*Assume (A0) holds. Assume there exist some* ${\left\{a_{i}\right\}}_{i},\;{\left\{R_{k}\right\}}_{k}$ *such that* $a_{i}\in {\mathbb{R}}^{s_{0}}$, $R_{k}\in {\mathbb{R}}^{s_{0}\times s_{0}}$ *and*

$$g\left(a_{i,0}^{T}R_{k,0}a_{j,0}\right)=g\left(a_{i}^{T}R_{k}a_{j}\right),\;\forall i,j,k.$$

*Then, there exists some invertible matrix* $C\in {\mathbb{R}}^{s_{0}\times s_{0}}$ *such that*

$$a_{i}=C^{-1}a_{i,0}\;\text{and}\;R_{k}=C^{T}R_{k,0}C.$$

To fix the nonsingular transformation indeterminacy, we adopt a specific constrained parameterization and focus on estimating ${\left\{a_{i}^{*}\right\}}_{i}$ and ${\left\{R_{k}^{*}\right\}}_{k}$ where

$$a_{i}^{*}={\left(A_{s_{0},0}^{-1}\right)}^{T}a_{i,0}\;\text{and}\;R_{k}^{*}=A_{s_{0},0}R_{k,0}A_{s_{0},0}^{T},$$

where $A_{s_{0},0}={\left[a_{1,0},\ldots ,a_{s_{0},0}\right]}^{T}$. Observe that

$$\left[a_{1}^{*},\ldots ,a_{s_{0}}^{*}\right]={\left(A_{s_{0},0}^{-1}\right)}^{T}\left[a_{1,0},\ldots ,a_{s_{0},0}\right]={\left(A_{s_{0},0}^{-1}\right)}^{T}A_{s_{0},0}^{T}=I_{s_{0}},$$

where ***I***_*s*_0__ stands for an *s*_0_ × *s*_0_ identity matrix. Therefore, the first $s_{0}\;a_{i}^{*}$’s are fixed as long as $A_{s_{0},0}$ is nonsingular. By Lemma 1, the parameters ${\left\{a_{i}^{*}\right\}}_{i}$ and ${\left\{R_{k}^{*}\right\}}_{k}$ are estimable.

From now on, we only consider the logistic link function for simplicity, i.e, g(*x*) = 1/{1 + exp(−*x*)}. Results for other link functions can be similarly discussed.

## 3. Model Selection

Parameters ${\left\{a_{i}^{*}\right\}}_{i=1}^{n}$ and ${\left\{R_{k}^{*}\right\}}_{k=1}^{K}$ can be estimated by maximizing the (conditional) log-likelihood function. Since we use the logistic link function, the log-likelihood is equal to

$$l_{n}\left(Y;{\left\{a_{i}\right\}}_{i},{\left\{R_{k}\right\}}_{k}\right)=\log\left(\prod_{ijk}\text{g}{\left(a_{i}^{T}R_{k}a_{j}\right)}^{Y_{ijk}}{\left\{1-\text{g}\left(a_{i}^{T}R_{k}a_{j}\right)\right\}}^{1-Y_{ijk}}\right)\;\;=\sum_{ijk}\left(Y_{ijk}\log\left\{\text{g}\left(a_{i}^{T}R_{k}a_{j}\right)\right\}+\left(1-Y_{ijk}\right)\log\left\{1-\text{g}\left(a_{i}^{T}R_{k}a_{j}\right)\right\}\right)\;\;=\sum_{ijk}\left(Y_{ijk}a_{i}^{T}R_{k}a_{j}-\log\left\{1+\exp\left(a_{i}^{T}R_{k}a_{j}\right)\right\}\right),$$

where the first equality is due to the conditional independence assumption.

We assume the number of latent factors *s*_0_ is fixed. For any $s\in \left\{1,\ldots ,s_{\max}\right\}$ here *s*_max_ is allowed to diverge with *n* and satisfies *s*_max_ ≥ *s*_0_, we define the following constrained maximum likelihood estimator

(3) $$\left({\left\{{\hat{a}}_{i}^{\left(s\right)}\right\}}_{i},{\left\{{\hat{R}}_{k}^{\left(s\right)}\right\}}_{k}\right)=\underset{\underset{\mathrm{vec}\left(R_{1}^{\left(s\right)}\right),\ldots ,\mathrm{vec}\left(R_{K}^{\left(s\right)}\right)\in \Theta_{r}^{\left(s\right)}}{a_{1}^{\left(s\right)},\ldots ,a_{n}^{\left(s\right)}\in \Theta_{a}^{\left(s\right)}}}{\text{arg max}}\;l_{n}\left(Y;{\left\{a_{i}^{\left(s\right)}\right\}}_{i},{\left\{R_{k}^{\left(s\right)}\right\}}_{k}\right),$$

(4) $$\text{subject to}\;\left[a_{1}^{\left(s\right)},\ldots ,a_{s}^{\left(s\right)}\right]=I_{s},$$

for some $\Theta_{a}^{\left(s\right)}\subseteq {\mathbb{R}}^{s},\;\Theta_{r}^{\left(s\right)}\subseteq {\mathbb{R}}^{s^{2}}$, where the vec(·) operator stacks the entries of a matrix into a column vector. To estimate the number of latent factors, we define the following likelihood-based information criteria

$$\text{IC}(s)=2l_{n}\left(Y;{\left\{{\hat{a}}_{i}^{\left(s\right)}\right\}}_{i},{\left\{{\hat{R}}_{k}^{\left(s\right)}\right\}}_{k}\right)-s\kappa (n,K),$$

for some penalty functions *κ*(·,·). The estimated number of latent factors is given by

(5) $$\hat{s}=\underset{s\in \left\{1,\ldots ,s_{\max}\right\}}{\text{arg max}}\;\mathrm{IC}(s).$$

In addition to the constraint in Equation 4, there exist many other constraints that would make the estimators identifiable. The choice of the identifiability constraints might affect the value of IC. However, it wouldn’t affect the value of $\hat{s}$. Detailed discussions can be found in Section A of the Appendix.

A major technical difficulty in establishing the consistency of IC is due to the nonconcavity of the objective function given in Equation 3. For any ${\left\{a_{j}\right\}}_{j\in \{1,\ldots ,n\}},{\left\{R_{k}\right\}}_{k\in \{1,\ldots ,K\}}$, let

$$\beta ={\left(a_{1}^{T},\ldots ,a_{n}^{T},\mathrm{vec}{\left(R_{1}\right)}^{T},\ldots ,\mathrm{vec}{\left(R_{K}\right)}^{T}\right)}^{T},$$

be the set of parameters.

For any $b_{1},\ldots ,b_{n}\in {\mathbb{R}}^{s},T_{1},\ldots ,T_{K}\in {\mathbb{R}}^{s\times s}$, we define

$$\zeta ={\left(b_{1}^{T},\ldots ,b_{n}^{T},\mathrm{vec}{\left(T_{1}\right)}^{T},\ldots ,\mathrm{vec}{\left(T_{K}\right)}^{T}\right)}^{T}.$$

With some calculations, we can show that

$$-\zeta^{T}\frac{\partial^{2}l_{n}}{\partial \beta \partial \beta^{T}}\zeta =\underset{I_{1}}{\underset{︸}{\sum_{ijk}\pi_{ijk}\left(1-\pi_{ijk}\right){\left(b_{i}^{T}R_{k}a_{j}+a_{i}^{T}R_{k}b_{j}+a_{i}^{T}T_{k}a_{j}\right)}^{2}}}\;\;+\underset{I_{2}}{\underset{︸}{\sum_{ijk}\left(\pi_{ijk}-Y_{ijk}\right)\left(2b_{i}^{T}R_{k}b_{j}+b_{i}^{T}T_{k}a_{j}+a_{i}^{T}T_{k}b_{j}\right)}},$$

where $\pi_{ijk}=\exp\left(a_{i}^{T}R_{k}a_{j}\right)/\left\{1+\exp\left(a_{i}^{T}R_{k}a_{j}\right)\right\}$. Here, *I*_1_ is nonnegative. However, *I*_2_ can be negative for some ***β*** and ***ζ***. Therefore, the negative Hessian matrix is not positive semidefinite and the likelihood function is not concave. As a result, ${\hat{a}}_{i}^{s_{0}}$ and ${\hat{R}}_{k}^{s_{0}}$ may not be consistent to ${\hat{a}}_{i}^{*}$ and ${\hat{R}}_{k}^{*}$, even with the identifiability constraints in Equation 4. Here, the presence of *I*_2_ is due to the bilinear formulation of the RESCAL model.

Let $\theta_{ijk}=a_{i}^{T}R_{k}a_{j}$. Notice that $l_{n}$ is concave in *θ*_*ijk*_, ∀*i, j, k*. This motivates us to consider the following pseudometric:

$$\;d\left({\left\{a_{i,1}^{\left(s_{1}\right)}\right\}}_{i},{\left\{R_{k,1}^{\left(s_{1}\right)}\right\}}_{k_{1}};{\left\{a_{i,2}^{\left(s_{2}\right)}\right\}}_{i},{\left\{R_{k,2}^{\left(s_{2}\right)}\right\}}_{k_{2}}\right)\;={\left\{\frac{1}{n^{2}K}\sum_{ijk}{\left({\left(a_{i,1}^{\left(s_{1}\right)}\right)}^{T}{\left(R_{k,1}^{\left(s_{1}\right)}\right)}^{T}a_{j,1}^{\left(s_{1}\right)}-{\left(a_{i,2}^{\left(s_{2}\right)}\right)}^{T}{\left(R_{k,2}^{\left(s_{2}\right)}\right)}^{T}a_{j,2}^{\left(s_{2}\right)}\right)}^{2}\right\}}^{1/2},$$

for any integers *s*_1_, *s*_2_ > 0 and $a_{i,1}^{\left(s_{1}\right)}\in {\mathbb{R}}^{s_{1}}$, $R_{k,1}^{\left(s_{1}\right)}\in {\mathbb{R}}^{s_{1}\times s_{1}}$, $a_{i,2}^{\left(s_{2}\right)}\in {\mathbb{R}}^{s_{2}}$, $R_{k,2}^{\left(s_{2}\right)}\in {\mathbb{R}}^{s_{2}\times s_{2}}$. Apparently, *d*(·,·) is nonnegative, symmetric and satisfies the triangle inequality. Below, we establish the convergence rate of

$$d\left({\left\{{\hat{a}}_{i}^{\left(s\right)}\right\}}_{i},{\left\{{\hat{R}}_{k}^{\left(s\right)}\right\}}_{k};{\left\{a_{i}^{*}\right\}}_{i},{\left\{R_{k}^{*}\right\}}_{k}\right).$$

We first introduce some notation. For any *s* > *s*_0_, we define

$$a_{i,0}^{\left(s\right)}=\{\begin{array}{ll} {\left({\left(a_{i,0}\right)}^{T},0_{s-s_{0}}^{T}\right)}^{T}, & i\notin \left\{s_{0}+1,\ldots ,s\right\},\\ ({\left(a_{i,0}\right)}^{T},\underset{i-s_{0}-1}{\underset{︸}{0,\ldots ,0,}}1,\underset{s-i}{\underset{︸}{0,\ldots ,0{)}^{T}}}, & i\in \left\{s_{0}+1,\ldots ,s\right\}, \end{array}$$

and

$$R_{k,0}^{\left(s\right)}=\left(\begin{array}{cc} R_{k,0} & O_{r,s-r}\\ O_{s-r,r} & O_{s-r,s-r} \end{array}\right),$$

where **0**_*q*_ denotes a *q*-dimensional zero vector and ***O***_*p,q*_ is an *p* × *q* zero matrix. With a slight abuse of notation, we write $a_{i,0}^{\left(s_{0}\right)}=a_{i,0}$ and $R_{k,0}^{\left(s_{0}\right)}=R_{k,0}$. Clearly, for any *s* ≥ *s*_0_, we have

$${\left(a_{i,0}^{\left(s\right)}\right)}^{T}R_{k,0}^{\left(s\right)}a_{j,0}^{\left(s\right)}=a_{i,0}^{T}R_{k,0}a_{j,0},\;\forall i,j,k,$$

and hence

$$\left({\left\{a_{i,0}^{\left(s\right)}\right\}}_{i},{\left\{R_{k,0}^{\left(s\right)}\right\}}_{k}\right)=\underset{{\left\{a_{i}^{\left(s\right)}\right\}}_{i},{\left\{R_{k}^{\left(s\right)}\right\}}_{k}}{\arg\max}El_{n}\left(Y;{\left\{a_{i}^{\left(s\right)}\right\}}_{i},{\left\{R_{k}^{\left(s\right)}\right\}}_{k}\right),$$

Let

$$a_{i}^{(s)*}={\left(A_{s,0}^{-1}\right)}^{T}a_{i,0}^{\left(s\right)}\;\text{and}\;R_{k}^{(s)*}=A_{s,0}R_{k,0}^{\left(s\right)}A_{s,0}^{T},$$

where $A_{s,0}={\left[a_{1,0}^{\left(s\right)},\ldots ,a_{s,0}^{\left(s\right)}\right]}^{T}$. When $A_{s_{0},0}$ is invertible, ***A***_*s*,0_’s are invertible for all *s* > *s*_0_. The defined ${\left\{a_{i}^{(s)*}\right\}}^{\prime }$’s satisfy the identifiability constraints in Equation 4 for all *s* ≥ *s*_0_. We make the following assumption.

(A1) Assume $a_{i}^{(s)*}\in \Theta_{a}^{\left(s\right)}$ and $\mathrm{vec}\left(R_{k}^{(s)*}\right)\in \Theta_{r}^{\left(s\right)}$, ∀i=1,…,n,k=1,…,K and *s*_0_ ≤ *s* ≤ *s*_max_. In addition, assume $\sup_{x\in \Theta_{a}^{\left(s\right)}}\|x{\|}_{2}\leq \omega_{a}$, $\sup_{y\in \Theta_{r}^{\left(s\right)}}\|y{\|}_{2}\leq \omega_{r}$ for some $\omega_{a},\omega_{r}>0$.

### Lemma 2.

*Assume (A1) holds*, $s_{\max}=o\{\sqrt{n/\log(nK)}\}$. *Then there exists some constant C*_0_ > 0 *such that the following event occurs with probability tending to* 1,

$$\underset{s\in \left\{s_{0},\ldots ,s_{\max}\right\}}{\max}\frac{1}{s^{2}}d^{2}\left({\left\{{\hat{a}}_{i}^{\left(s\right)}\right\}}_{i},{\left\{{\hat{R}}_{k}^{\left(s\right)}\right\}}_{k};{\left\{a_{i}^{*}\right\}}_{i},{\left\{R_{k}^{*}\right\}}_{k}\right)\leq \frac{\exp\left(2\omega_{a}^{2}\omega_{r}\right)(n+K)(\log n+\log K)}{n^{2}K}.$$

Under the condition $s_{\max}=o\{\sqrt{n/\log(nK)}\}$, we have that

$$\frac{s_{\max}^{2}(n+K)(\log n+\log K)}{n^{2}K}\leq \frac{s_{\max}^{2}(2n\log n+2K\log K)}{n^{2}K}\leq \frac{2s_{\max}^{2}\log n}{n}+\frac{2s_{\max}^{2}\log K}{n^{2}}=o(1).$$

When *ω*_*a*_ and *ω*_*r*_ are bounded, it follows that

$$\underset{s\in \left\{s_{0},\ldots ,s_{\max}\right\}}{\max}d^{2}\left({\left\{{\hat{a}}_{i}^{\left(s\right)}\right\}}_{i},{\left\{{\hat{R}}_{k}^{\left(s\right)}\right\}}_{k};{\left\{a_{i}^{*}\right\}}_{i},{\left\{R_{k}^{*}\right\}}_{k}\right)\leq \frac{s_{\max}^{2}\exp\left(2\omega_{a}^{2}\omega_{r}\right)(n+K)(\log n+\log K)}{n^{2}K}=o(1).$$

Hence, ${\left\{{\hat{a}}_{i}^{\left(s\right)}\right\}}_{i}$ and ${\left\{{\hat{R}}_{k}^{\left(s\right)}\right\}}_{k}$ are consistent under the pseudometric *d* for all overfitted models. On the contrary, for underfitted models, we require the following conditions.

(A2) Assume there exists some constant $\bar{c}>0$ such that $\lambda_{\min}\left(A_{0}^{T}A_{0}\right)\geq n\bar{c}$.

(A3) Let $\bar{K}=\lambda_{\min}\left(\sum_{k=1}^{K}R_{k,0}^{T}R_{k,0}\right)$. Assume ${\lim\;\inf}_{n}\bar{K}>0$.

### Lemma 3.

*Assume (A2) and (A3) hold. The for any* $s\in \left\{1,2,\ldots ,s_{0}-1\right\}$, *we have*

$$d^{2}\left({\left\{{\hat{a}}_{i}^{\left(s\right)}\right\}}_{i},{\left\{{\hat{R}}_{k}^{\left(s\right)}\right\}}_{k};{\left\{a_{i}^{*}\right\}}_{i},{\left\{R_{k}^{*}\right\}}_{k}\right)\geq \frac{{\bar{c}}^{2}\bar{K}}{K},$$

*where* $\bar{c}$ *and* $\bar{K}$ *are defined in (A2) and (A3), respectively*.

Assumption (A3) holds if there exists some $k_{0}\in \{1,\ldots ,K\}$ such that

$${\lim\;\inf}_{n}\lambda_{\min}\left(R_{k_{0},0}R_{k_{0},0}^{T}\right)>0.$$

When $\bar{K}\geq c^{\prime }K$ for some constant *c*^′^ > 0, it follows from Lemma 3 that

$$\underset{n}{\lim\inf}d\left({\left\{{\hat{a}}_{i}^{\left(s\right)}\right\}}_{i},{\left\{{\hat{R}}_{k}^{\left(s\right)}\right\}}_{k};{\left\{a_{i}^{*}\right\}}_{i},{\left\{R_{k}^{*}\right\}}_{k}\right)>0.$$

Based on these results, we establish the consistency of $\hat{s}$ defined in Equation 5 below. For any sequences {*a*_*n*_} and {*b*_*n*_}, we write *a*_*n*_ ~ *b*_*n*_ if there exist some universal constants *c*_1_, *c*_2_ > 0 such that *c*_1_*a*_*n*_ ≤ *b*_*n*_ ≤ *c*_2_*a*_*n*_.

#### Theorem 1.

*Assume (A1)-(A3) hold*, $K\sim n^{l_{0}}$ *or some* $0\leq l_{0}\leq 1,s_{\max}=o\{\sqrt{n/\log(nK)}\},\lim\inf_{n}n^{\left(1-l_{0}\right)/2}\bar{K}\geq \exp\left(2\omega_{a}^{2}\omega_{r}\right)\sqrt{\log n}$. *Assume κ*(*n, K*) *satisfies*

(6) $$s_{\max}\exp\left(\omega_{a}^{2}\omega_{r}\right)(n+K)(\log n+\log K)\ll \kappa (n,K)\ll \frac{n^{2}\bar{K}}{\exp\left(\omega_{a}^{2}\omega_{r}\right)}.$$

*Then, we have* $\mathrm{Pr}\left(\hat{s}=s_{0}\right)\to 1$ *where $\hat{s}$ is defined in Equation 5*.

Let $c(n,K)=\kappa (n,K){\left(n+K\right)}^{-1}{\left(\log n+\log K\right)}^{-1}$. When $s_{\max},\omega_{a},\omega_{r}$ are bounded, it follows from Theorem 1 that IC is consistent provided that c(n,K)→∞ and $c(n,K)=o(n\bar{K}/\log n)$. Define

$$\tau_{\alpha }(n,K)=\frac{{\left(n+K\right)}^{\alpha }}{\max^{\alpha }(n,K)},$$

for some *α* ≥ 0. Note that

(7) $$1\leq \tau_{\alpha }(n,K)\leq 2^{\alpha }.$$

Consider the following criteria:

(8) $${\text{IC}}_{\alpha }(s)=2l_{n}\left(Y;{\left\{{\hat{a}}_{i}^{\left(s\right)}\right\}}_{i},{\left\{{\hat{R}}_{k}^{\left(s\right)}\right\}}_{k}\right)-s\tau_{\alpha }(n,K)(n+K)(\log n+\log K)\log\{\log(nK)\}.$$

Note that the term log{log(nK)} satisfies that log{log(nK)}→∞ and log{log(nK)}=o(n/logn). It follows from Equation 7 and Theorem 1 that IC_*α*_ is consistent for all *α* ≥ 0. When *α* > 0, the term $\tau_{\alpha }(n,K)$ adjust the model complexity penalty upwards. We notice that Bai and Ng (2002) used a similar finite sample correction term in their proposed information criteria for approximate factor models. Our simulation studies show that such adjustment is essential to achieve selection consistency for large *K*.

Conditions (A1) and (A2) are directly imposed on the realizations of $a_{1,0},\ldots ,a_{n,0}$. In Section B.2 and B.3, we consider an asymptotic framework where $a_{1,0},\ldots ,a_{n,0}$ are i.i.d according to some distribution function and show (A1) and (A2) hold with probability tending to 1. Therefore, under this framework, we still have $\mathrm{Pr}\left(\hat{s}=s_{0}\right)\to 1$. The consistency of our information criterion remains unchanged.

Observe that we have a total of *n* × *n* × *K* = *n*^2^*K* observations. Consider the following BIC-type criterion:

(9) $$\text{BIC}(s)=2l_{n}\left(Y;{\left\{{\hat{a}}_{i}^{\left(s\right)}\right\}}_{i},{\left\{{\hat{R}}_{k}^{\left(s\right)}\right\}}_{k}\right)-s\log\left(n^{2}K\right).$$

The model complexity penalty in BIC satisfies

$$\log\left(n^{2}K\right)=2\log n+\log K\ll (n+K)(\log n+\log K).$$

Hence, it does not meet Condition (6) in Theorem 1. As a result, BIC may fail to identity the true model. As shown in our simulation studies, BIC will choose overfitted models and is not selection consistent.

## 4. Numerical Experiments

This section is organized as follows. In Section 4.1, we introduce our algorithm for computing the maximum likelihood estimators of a logistic RESCAL model. Simulation studies are presented in Section 4.2. In Section 4.3, we apply the proposed information criteria to a real dataset.

### 4.1. Implementation

In this section, we propose an algorithm for computing ${\left\{{\hat{a}}_{i}^{\left(s\right)}\right\}}_{i}$ and ${\left\{{\hat{R}}_{k}^{\left(s\right)}\right\}}_{k}$. The algorithm is based upon a 3-block alternating direction method of multipliers (ADMM). Set $\Theta_{a}^{\left(s\right)}={\mathbb{R}}^{s}$, $\Theta_{r}^{\left(s\right)}={\mathbb{R}}^{s\times s}$, $\left[a_{1}^{\left(s\right)},\ldots ,a_{s}^{\left(s\right)}\right]=I_{s}$, ${\hat{a}}_{i}^{\left(s\right)}$’s and ${\hat{R}}_{k}^{\left(s\right)}$’s are defined by

(10) $$\left({\left\{{\hat{a}}_{i}^{\left(s\right)}\right\}}_{i=(s+1)}^{n},{\left\{{\hat{R}}_{k}^{\left(s\right)}\right\}}_{k}\right)=\underset{{\left\{a_{i}^{\left(s\right)}\right\}}_{i=s+1}^{n},{\left\{R_{k}^{\left(s\right)}\right\}}_{k}}{\arg\max}l_{n}\left(Y;{\left\{a_{i}^{\left(s\right)}\right\}}_{i},{\left\{R_{k}^{\left(s\right)}\right\}}_{k}\right),$$

where

$$l_{n}\left(Y;{\left\{a_{i}^{\left(s\right)}\right\}}_{i},{\left\{R_{k}^{\left(s\right)}\right\}}_{k}\right)=\sum_{ijk}\left(Y_{ijk}{\left(a_{i}^{\left(s\right)}\right)}^{T}R_{k}^{\left(s\right)}a_{j}^{\left(s\right)}-\log\left[1+\exp\left\{{\left(a_{i}^{\left(s\right)}\right)}^{T}R_{k}^{\left(s\right)}a_{j}^{\left(s\right)}\right\}\right]\right).$$

For any $b_{1},\ldots ,b_{n}\in {\mathbb{R}}^{s}$, define

$${\bar{l}}_{n}\left(Y;{\left\{a_{i}^{\left(s\right)}\right\}}_{i},{\left\{R_{k}^{\left(s\right)}\right\}}_{k},{\left\{b_{i}^{\left(s\right)}\right\}}_{i}\right)=\sum_{ijk}\left(Y_{ijk}{\left(a_{i}^{\left(s\right)}\right)}^{T}R_{k}^{\left(s\right)}b_{j}^{\left(s\right)}-\log\left[1+\exp\left\{{\left(a_{i}^{\left(s\right)}\right)}^{T}R_{k}^{\left(s\right)}b_{j}^{\left(s\right)}\right\}\right]\right).$$

Fix $\left[b_{1}^{\left(s\right)},\ldots ,b_{s}^{\left(s\right)}\right]=I_{s}$, the optimization problem in Equation 10 is equivalent to

$$\left({\left\{{\hat{a}}_{i}^{\left(s\right)}\right\}}_{i=s+1}^{n},{\left\{{\hat{R}}_{k}^{\left(s\right)}\right\}}_{k},{\left\{{\hat{b}}_{i}^{\left(s\right)}\right\}}_{i=s+1}^{n}\right)=\underset{\begin{array}{c} {\left\{a_{i}^{\left(s\right)}\right\}}_{i=s+1}^{n},{\left\{R_{k}^{\left(s\right)}\right\}}_{k}\\ {\left\{b_{i}^{\left(s\right)}\right\}}_{i=s+1}^{n} \end{array}}{\arg\max}{\bar{l}}_{n}\left(Y;{\left\{a_{i}^{\left(s\right)}\right\}}_{i},{\left\{R_{k}^{\left(s\right)}\right\}}_{k},{\left\{b_{i}^{\left(s\right)}\right\}}_{i}\right)$$

$$\text{subject to}\;a_{i}^{\left(s\right)}=b_{i}^{\left(s\right)},\forall i=s+1,\ldots ,n.$$

We then derive its augmented Lagrangian, which gives us

$$\;L_{\rho }\left({\left\{a_{i}^{\left(s\right)}\right\}}_{i=s+1}^{n},{\left\{R_{k}^{\left(s\right)}\right\}}_{k},{\left\{b_{i}^{\left(s\right)}\right\}}_{i=s+1}^{n},{\left\{v_{i}^{\left(s\right)}\right\}}_{i=s+1}^{n}\right)\;=-{\bar{l}}_{n}\left(Y;{\left\{a_{i}^{\left(s\right)}\right\}}_{i},{\left\{R_{k}^{\left(s\right)}\right\}}_{k},{\left\{b_{i}^{\left(s\right)}\right\}}_{i}\right)+\sum_{i=s+1}^{n}\rho {\left(a_{i}^{\left(s\right)}-b_{i}^{\left(s\right)}\right)}^{T}v_{i}^{\left(s\right)}+\sum_{i=s+1}^{n}\frac{\rho }{2}{\left\|a_{i}^{\left(s\right)}-b_{i}^{\left(s\right)}\right\|}_{2}^{2},$$

where *ρ* > 0 is a penalty parameter and, $v_{s+1}^{\left(s\right)},\ldots ,v_{n}^{\left(s\right)}\in {\mathbb{R}}^{s}$.

Applying dual descent method yields the following steps, with *l* denotes the iteration number:

(11) $${\left\{a_{i,l+1}^{\left(s\right)}\right\}}_{i=s+1}^{n}=\underset{{\left\{a_{i}^{\left(s\right)}\right\}}_{i=s+1}^{n}}{\arg\min}L_{\rho }\left({\left\{a_{i}^{\left(s\right)}\right\}}_{i=s+1}^{n},{\left\{R_{k,l}^{\left(s\right)}\right\}}_{k},{\left\{b_{i,l}^{\left(s\right)}\right\}}_{i=s+1}^{n},{\left\{v_{i,l}^{\left(s\right)}\right\}}_{i=s+1}^{n}\right),$$

(12) $${\left\{R_{k,l+1}^{\left(s\right)}\right\}}_{k=1}^{K}=\underset{{\left\{R_{k}^{\left(s\right)}\right\}}_{k=1}^{K}}{\arg\min}L_{\rho }\left({\left\{a_{i,l+1}^{\left(s\right)}\right\}}_{i=s+1}^{n},{\left\{R_{k}^{\left(s\right)}\right\}}_{k},{\left\{b_{i,l}^{\left(s\right)}\right\}}_{i=s+1}^{n},{\left\{v_{i,l}^{\left(s\right)}\right\}}_{i=s+1}^{n}\right),$$

(13) $${\left\{b_{i,l+1}^{\left(s\right)}\right\}}_{i=s+1}^{n}=\underset{{\left\{b_{i}^{\left(s\right)}\right\}}_{i=s+1}^{n}+1}{\arg\min}L_{\rho }\left({\left\{a_{i,l+1}^{\left(s\right)}\right\}}_{i=s+1}^{n},{\left\{R_{k,l+1}^{\left(s\right)}\right\}}_{k},{\left\{b_{i}^{\left(s\right)}\right\}}_{i=s+1}^{n},{\left\{v_{i,l}^{\left(s\right)}\right\}}_{i=s+1}^{n}\right),$$

$$v_{i,l+1}^{\left(s\right)}=v_{i,l}^{\left(s\right)}+a_{i,l}^{\left(s\right)}-b_{i,l}^{\left(s\right)},\;\forall i=s+1,\ldots ,n.$$

Let us examine Equation 11–13 in more details. In Equation 11, we rewrite the objective function as

$$\;L_{\rho }\left({\left\{a_{i}^{\left(s\right)}\right\}}_{i=s+1}^{n},{\left\{R_{k,l}^{\left(s\right)}\right\}}_{k},{\left\{b_{i,l}^{\left(s\right)}\right\}}_{i=s+1}^{n},{\left\{v_{i,l}^{\left(s\right)}\right\}}_{i=s+1}^{n}\right)\;=\sum_{i=s+1}^{n}\{\sum_{j,k}\left(\log\left[1+\exp\left\{{\left(a_{i}^{\left(s\right)}\right)}^{T}R_{k,l}^{\left(s\right)}b_{j,l}^{\left(s\right)}\right\}\right]-Y_{ijk}{\left(a_{i}^{\left(s\right)}\right)}^{T}R_{k,l}^{\left(s\right)}b_{j,l}^{\left(s\right)}\right)\;+\rho {\left(a_{i}^{\left(s\right)}-b_{i,l}^{\left(s\right)}\right)}^{T}v_{i,l}^{\left(s\right)}+\frac{\rho }{2}{\left\|a_{i}^{\left(s\right)}-b_{i,l}^{\left(s\right)}\right\|}_{2}^{2}\}.$$

Note that *L*_*ρ*_ can be represented as a separable sum of functions. As a result, $a_{i,l+1}^{\left(s\right)}$‘s can be solved in parallel. More specifically, we have

$$a_{i,l+1}^{\left(s\right)}=\underset{a^{\left(s\right)}}{\arg\min}\{\sum_{j,k}\left(\log\left[1+\exp\left\{{\left(a_{i}^{\left(s\right)}\right)}^{T}R_{k,l}^{\left(s\right)}b_{j,l}^{\left(s\right)}\right\}\right]-Y_{ijk}{\left(a_{i}^{\left(s\right)}\right)}^{T}R_{k,l}^{\left(s\right)}b_{j,l}^{\left(s\right)}\right)\;\;+\rho {\left(a_{i}^{\left(s\right)}-b_{i,l}^{\left(s\right)}\right)}^{T}v_{i,l}^{\left(s\right)}+\frac{\rho }{2}{\left\|a_{i}^{\left(s\right)}-b_{i,l}^{\left(s\right)}\right\|}_{2}^{2}\}.$$

Hence, each $a_{i,l+1}^{\left(s\right)}$ can be computed by solving a ridge type logistic regression with responses ${\left\{Y_{ijk}\right\}}_{j,k}$ and covariates ${\left\{R_{k,l}^{\left(s\right)}b_{j,l}^{\left(s\right)}\right\}}_{j,k}$.

In Equation 12, each $R_{k,l+1}^{\left(s\right)}$ can be independently updated by solving a logistic regression with responses ${\left\{Y_{ijk}\right\}}_{i,j}$ and covariates $b_{j,l}^{\left(s\right)}⊗a_{i,l+1}^{\left(s\right)}$, i.e,

$$\mathrm{vec}\left(R_{k,l+1}^{\left(s\right)}\right)=\underset{r_{k}^{\left(s\right)}\in {\mathbb{R}}^{s^{2}}}{\arg\min}\sum_{ij}\left\{\log\left(1+\exp\left[\left\{{\left(b_{j,l}^{\left(s\right)}\right)}^{T}⊗{\left(a_{i,l+1}^{\left(s\right)}\right)}^{T}\right\}r_{k}^{\left(s\right)}\right]\right)-Y_{ijk}\left\{{\left(b_{j,l}^{\left(s\right)}\right)}^{T}⊗{\left(a_{i,l+1}^{\left(s\right)}\right)}^{T}\right\}r_{k}^{\left(s\right)}\right\},$$

where ⊗ denotes the Kronecker product.

Similar to Equation 11, each $b_{i,l+1}^{\left(s\right)}$ in Equation 13 can be independently computed by solving a ridge type regression with responses ${\left\{Y_{ijk}\right\}}_{j,k}$ and covariates ${\left\{{\left(R_{k,l+1}^{\left(s\right)}\right)}^{T}a_{j,l+1}^{\left(s\right)}\right\}}_{j,k}$.

Using similar arguments in Theorem 2 in Wang et al. (2017), we can show that the proposed 3-block ADMM algorithm converges for any sufficiently large *ρ*. In our implementation, we set *ρ* = *nK*/2. To guarantee global convergence, we randomly generate multiple initial estimators and solve the optimization problem multiple times based on these initial values.

### 4.2. Simulations

We simulate ${\left\{Y_{ijk}\right\}}_{ijk}$ from the following model:

$$\mathrm{Pr}\left(Y_{ijk}=1|{\left\{a_{i}\right\}}_{i},{\left\{R_{k}\right\}}_{k}\right)=\frac{\exp\left(a_{i}^{T}R_{k}a_{j}\right)}{1+\exp\left(a_{i}^{T}R_{k}a_{j}\right)},$$

$$a_{1},a_{2},\ldots ,a_{n}\overset{iid}{\sim }N(0,1),$$

$$R_{1}=R_{2}=\cdots =R_{K}=\mathrm{diag}(\underset{s_{0}}{\underset{︸}{1,-1,1,-1,\ldots ,1,-1}})$$

where *N*(0, 1) stands for a standard normal random variable and $\mathrm{diag}\left(v_{1},\ldots ,v_{q}\right)$ denotes a *q* × *q* diagonal matrix with the *j*th element equal to *v*_*j*_.

We consider six simulation settings. In the first three settings, we fix *K* = 3 and set *n* = 100,150 and 200, respectively. In the last three settings, we increase *K* to 10, 20, 50, and set *n* = 50. In each setting, we further consider three scenarios, by setting *s*_0_ = 2, 4 and 8. Let *s*_max_ = 12. The ADMM algorithm proposed in Section 4.1 is implemented in R. Some subroutines of the algorithm are written in C with the GNU Scientific Library (GSL, Galassi et al., 2015) to facilitate the computation. We compare the proposed IC_*α*_ (see Equation 8) with the BIC-type criterion (see Equation 9). In IC_*α*_, we set *α* = 0, 0.5 and 1. Note that when *α* = 0, we have

$$\tau_{\alpha }(n,K)=\frac{{\left(n+K\right)}^{\alpha }}{\max^{\alpha }(n,K)}=1.$$

Reported in Table 1 and 2 are the percentage of selecting the true models (TP) and the average of $\hat{s}$ selected by IC_0_, IC_0.5_, IC_1_ and BIC over 100 replications.

It can be seen from Table 1 and 2 that BIC fails in all settings. It always selects overfitted models. On the contrary, the proposed information criteria are consistent for most of the settings. For example, under settings where *s*_0_ = 2 or 4, TPs of IC_0_, IC_0.5_ and IC_1_ are larger than or equal to 93%. When *s*_0_ = 8, expect for the last setting, TPs of the proposed information criteria are no less than 60% for all cases.

IC_0_, IC_0.5_ and IC_1_ perform very similarly for small *K*. In the first three settings, TPs of these three information criteria are nearly the same for all cases. However, IC_0.5_ and IC_1_ are more robust than IC_0_ for large *K*. This can be seen in the last scenario of Setting 6, where the TP of IC_0_ is no more than 20%. Besides, in the last two settings, TP of IC_0_ is smaller than IC_0.5_ and IC_1_ for all cases. These differences are due to the finite sample correction term $\tau_{\alpha }(n,K)$. As commented before, $\tau_{0.5}(n,K)$ and $\tau_{1}(n,K)$ increase the model complexity penalty term in IC_0.5_ and IC_1_ to avoid overfitting for large *K*.

In Section D of the Appendix, we examine the performance of our proposed information criteria under the scenario where $a_{1},a_{2},\ldots ,a_{n}\overset{iid}{\sim }N\left(0,{\left\{0.5^{\left|i-j\right|}\right\}}_{i,j=1,\ldots ,s_{0}}\right)$. Results are similar to those presented at Table 1 and 2.

### 4.3. Real Data Experiments

In this section, we apply the proposed information criteria to the “Social Evolution” dataset (Madan et al., 2012). This dataset comes from MIT’s Human Dynamics Laboratory. It tracks everyday life of a whole undergraduate MIT dormitory from October 2008 to May 2009. We use the survey data, resulting in *n* = 84 participants and *K* = 5 binary relations. The five relations are: close relationship, political discussion, social interaction and two social media interaction.

We compute ${\left\{{\hat{a}}_{i}^{\left(s\right)}\right\}}_{i}\;\text{and}\;{\left\{{\hat{R}}_{k}^{\left(s\right)}\right\}}_{k}$ for s={1,…,12} and select the number of latent factors using the proposed information criteria and BIC. It turns out that IC_0_, IC_0.5_ and IC_1_ all suggest the presence of 9 factors. In contrast, BIC selects 12 factors. To further evaluate the number of latent factors selected by the proposed information criteria, we consider the following cross-validation procedure. For any s∈[1,…,12], we randomly select 80% of the observations and estimate ${\left\{{\hat{a}}_{i}^{\left(s\right)}\right\}}_{i}$ and $\left\{{\hat{R}}_{k}^{\left(s\right)}\right\}$ by maximizing the observed likelihood function based on these training samples. Then we compute

$${\hat{\pi }}_{ijk}=\frac{\exp\left\{{\left({\hat{a}}_{i}^{\left(s\right)}\right)}^{T}{\hat{R}}_{k}^{\left(s\right)}{\hat{a}}_{j}^{\left(s\right)}\right\}}{1+\exp\left\{{\left({\hat{a}}_{i}^{\left(s\right)}\right)}^{T}{\hat{R}}_{k}^{\left(s\right)}{\hat{a}}_{j}^{\left(s\right)}\right\}}.$$

Based on these predicted probabilities, we calculate the area under the precision-recall curve (AUC) on the remaining 20% testing samples.

Reported in Table 3 are the AUC scores averaged over 100 replications. For any s∈{1,…,12}, we denoted by AUC_*s*_ the corresponding AUC score. It can be seen from Table 3 that AUC_*s*_ first increases and then decreases as *s* increases. The maximum AUC score is achieved at *s* = 10. Observe that AUC_9_ is very close to AUC_10_, and it is larger than the remaining AUC scores. This demonstrates that the proposed information criteria select less latent factors while achieve better or similar link prediction results when compared to BIC.

## 5. Discussion

In this paper, we propose information criteria for selecting the number of latent factors in the RESCAL tensor factorization model and prove their model selection consistency. Although we focus on the logistic RESCAL model, the proposed information criteria can be applied to general tensor factorization models. More specifically, consider the following class of models:

(14) $$Y_{ijk}=\text{g}\left(a_{i,0}^{T}R_{k,0}b_{j,0}\right)+e_{ijk},\;\forall i,j\in \{1,\ldots ,n\},\;k\in \{1,\ldots ,K\},$$

with any of (or without) the following constraints:

(C1) $R_{k,0}$ is diagonal;

(C2) $a_{i,0}=b_{i,0}$ for i∈{1,…,n},

for some strictly increasing function g, $a_{i,0},b_{i,0}\in {\mathbb{R}}^{s_{0}},R_{k,0}\in {\mathbb{R}}^{s_{0}\times s_{0}}$ and some mean zero random errors ${\left\{e_{ijk}\right\}}_{ijk}$.

As commented in Section 2.1, such representation includes the RESCAL, CP and TUCKER-2 models. Specifically, it reduces to the TUCKER-2 model by setting g to be the identity function. If further (C1) holds, then the model in Equation 14 reduces to the CP model. When (C2) holds, it corresponds to the RESCAL model. Consider the following information criteria,

$$\text{IC}(s)=l_{n}\left(Y;{\left\{{\hat{a}}_{i}\right\}}_{i},{\left\{{\hat{R}}_{k}\right\}}_{k},{\left\{{\hat{b}}_{i}\right\}}_{i}\right)-s\kappa (n,K),$$

where $l_{n}$ stands for the likelihood function and ${\hat{a}}_{i},{\hat{R}}_{k},{\hat{b}}_{i}$ are the corresponding (constrained) MLEs. Similar to Theorem 1, we can show that with some properly chosen κ(n,K), IC is consistent under this general setting.

Currently, we assume the tensor ***Y*** is completely observed. When some of the *Y*_*ijk*_’s are missing, we can calculate ${\hat{a}}_{i}^{\left(s\right)}$’s and ${\hat{R}}_{k}^{\left(s\right)}$’s by maximizing the following observed likelihood function

$$\underset{\begin{array}{c} a_{1}^{\left(s\right)},\ldots ,a_{n}^{\left(a\right)}\in \Theta_{a}^{\left(s\right)}\\ \mathrm{vec}\left(R_{1}^{\left(s\right)}\right),\ldots ,\mathrm{vec}\left(R_{K}^{\left(s\right)}\right)\in \Theta_{R}^{\left(s\right)} \end{array}}{\arg\max}\sum_{(i,j,k)\in N_{o}{}_{bs}}\left(Y_{ijk}{\left(a_{i}^{\left(s\right)}\right)}^{T}R_{k}^{\left(s\right)}a_{j}^{\left(s\right)}-\log\left[1+\exp\left\{{\left(a_{i}^{\left(s\right)}\right)}^{T}R_{k}^{\left(s\right)}a_{j}^{\left(s\right)}\right\}\right]\right),$$

$$\;\text{subject to}\;\left[a_{1}^{\left(s\right)},\ldots ,a_{s}^{\left(s\right)}\right]=I_{s},$$

where *N*_*obs*_ denotes the set of the observed responses. The above optimization problem can also be solved by a 3-block ADMM algorithm. Define the following class of information criteria,

$${\text{IC}}_{obs}(s)=\sum_{(i,j,k)\in N_{obs}}\left(Y_{ijk}{\left({\hat{a}}_{i}^{\left(s\right)}\right)}^{T}{\hat{R}}_{k}^{\left(s\right)}{\hat{a}}_{j}^{\left(s\right)}-\log\left[1+\exp\left\{{\left({\hat{a}}_{i}^{\left(s\right)}\right)}^{T}{\hat{R}}_{k}^{\left(s\right)}{\hat{a}}_{j}^{\left(s\right)}\right\}\right]\right)-\hat{p}s\kappa (n,K),$$

where $\hat{p}=\left|N_{obs}\right|/\left(n^{2}K\right)$ denotes the percentage of observed responses. Consistency of IC_*obs*_ can be similarly studied.

## Figures and Tables

**Table 1:** Simulation results for Setting I, II and III (standard errors in parenthesis)

	*s*_0_ = 2		*s*_0_ = 4		*s*_0_ = 8	
*n* = 100, *K* = 3	TP	$\hat{s}$	TP	$\hat{s}$	TP	$\hat{s}$
IC_0_	0.97 (0.02)	2.03 (0.02)	0.97 (0.02)	4.03 (0.02)	0.90(0.03)	7.90 (0.03)
IC_0.5_	0.97 (0.02)	2.03 (0.02)	0.98 (0.01)	4.02 (0.01)	0.90(0.03)	7.90 (0.03)
IC_1_	0.97 (0.02)	2.03 (0.02)	0.98 (0.01)	4.02 (0.01)	0.89(0.03)	7.89 (0.03)
BIC	0.00 (0.00)	11.99 (0.01)	0.00 (0.00)	12.00 (0.00)	0.00 (0.00)	11.99 (0.01)

*n* = 150, *K* = 3	TP	$\hat{s}$	TP	$\hat{s}$	TP	$\hat{s}$
IC_0_	0.99 (0.01)	2.01 (0.01)	0.97 (0.02)	4.03 (0.02)	0.96(0.02)	8.04 (0.02)
IC_0.5_	0.99 (0.01)	2.01 (0.01)	0.97 (0.02)	4.03 (0.02)	0.96(0.02)	8.04 (0.02)
IC_1_	0.99 (0.01)	2.01 (0.01)	0.97 (0.02)	4.03 (0.02)	0.96(0.02)	8.04 (0.02)
BIC	0.00 (0.00)	12.00 (0.00)	0.00 (0.00)	12.00 (0.00)	0.00 (0.00)	11.98 (0.01)

*n* = 200, *K* = 3	TP	$\hat{s}$	TP	$\hat{s}$	TP	$\hat{s}$
IC_0_	0.99 (0.01)	2.01 (0.01)	0.95 (0.02)	4.05 (0.02)	0.95(0.02)	8.05 (0.02)
IC_0.5_	0.99 (0.01)	2.01 (0.01)	0.95 (0.02)	4.05 (0.02)	0.95(0.02)	8.05 (0.02)
IC_1_	0.99 (0.01)	2.01 (0.01)	0.95 (0.02)	4.05 (0.02)	0.95(0.02)	8.05 (0.02)
BIC	0.00 (0.00)	12.00 (0.00)	0.00 (0.00)	11.99 (0.01)	0.00 (0.00)	11.98 (0.01)

**Table 2:** Simulation results for Setting IV, V and VI (standard errors in parenthesis)

	*s*_0_ = 2		*s*_0_ = 4		*s*_0_ = 8	
*n* = 50, *K* = 10	TP	$\hat{s}$	TP	$\hat{s}$	TP	$\hat{s}$
IC_0_	1.00 (0.00)	2.00 (0.00)	0.97 (0.02)	4.03 (0.02)	0.69(0.05)	7.91 (0.06)
IC_0.5_	1.00 (0.00)	2.00 (0.00)	0.97 (0.02)	4.03 (0.02)	0.66(0.05)	7.75 (0.06)
IC_1_	1.00 (0.00)	2.00 (0.00)	0.98 (0.01)	4.02 (0.01)	0.60(0.05)	7.62 (0.06)
BIC	0.00 (0.00)	11.81 (0.06)	0.00 (0.00)	11.60 (0.06)	0.01 (0.01)	11.67 (0.07)

*n* = 50, *K* = 20	TP	$\hat{s}$	TP	$\hat{s}$	TP	$\hat{s}$
IC_0_	0.97 (0.02)	2.03 (0.02)	0.95 (0.02)	4.05 (0.02)	0.73(0.04)	8.46 (0.10)
IC_0.5_	0.97 (0.02)	2.03 (0.02)	0.98 (0.01)	4.02 (0.01)	0.87(0.03)	8.09 (0.03)
IC_1_	0.98 (0.01)	2.02 (0.02)	1.00 (0.00)	4.00 (0.00)	0.79(0.04)	7.99 (0.05)
BIC	0.00 (0.00)	12.00 (0.00)	0.00 (0.00)	11.92 (0.03)	0.00 (0.00)	11.99 (0.01)

*n* = 50, *K* = 50	TP	$\hat{s}$	TP	$\hat{s}$	TP	$\hat{s}$
IC_0_	0.98 (0.01)	2.02 (0.01)	0.93 (0.03)	4.07 (0.03)	0.17(0.04)	11.24 (0.15)
IC_0.5_	0.99 (0.01)	2.01 (0.01)	0.97 (0.02)	4.03 (0.02)	0.76(0.04)	8.24 (0.05)
IC_1_	1.00 (0.00)	2.00 (0.00)	0.98 (0.01)	4.02 (0.01)	0.79(0.04)	7.99 (0.05)
BIC	0.00 (0.00)	12.00 (0.00)	0.00 (0.00)	12.00 (0.00)	0.00 (0.00)	11.99 (0.01)

**Table 3:** AUC scores

*s*	1	2	3	4	5	6	7	8	9	10	11	12
AUC	0.7201	0.8341	0.8952	0.9095	0.9257	0.9364	0.9444	0.9486	0.9513	0.9518	0.9485	0.9467

**Table 4:** Simulation results for Setting I, II and III (standard errors in parenthesis)

	*s*_0_ = 2		*s*_0_ = 4		*s*_0_ = 6	
*n* = 100, *K* = 3	TP	$\hat{s}$	TP	$\hat{s}$	TP	$\hat{s}$
IC_0_	1.00(0.00)	2.00(0.00)	0.96(0.02)	3.98(0.02)	0.88(0.03)	5.87(0.04)
IC_0.5_	1.00(0.00)	2.00(0.00)	0.96(0.02)	3.98(0.02)	0.88(0.03)	5.87(0.04)
IC_1_	1.00(0.00)	2.00(0.00)	0.96(0.02)	3.98(0.02)	0.85(0.04)	5.81(0.05)
BIC	0.00(0.00)	11.98(0.01)	0.00(0.00)	11.99(0.01)	0.00(0.00)	12.00(0.00)

*n* = 150, *K* = 3	TP	$\hat{s}$	TP	$\hat{s}$	TP	$\hat{s}$
IC_0_	1.00(0.00)	2.00(0.00)	0.97(0.02)	4.03(0.02)	0.94(0.02)	6.04(0.02)
IC_0.5_	1.00(0.00)	2.00(0.00)	0.97(0.02)	4.03(0.02)	0.94(0.02)	6.04(0.02)
IC_1_	1.00(0.00)	2.00(0.00)	0.97(0.02)	4.03(0.02)	0.94(0.02)	6.04(0.02)
BIC	0.00(0.00)	12.00(0.00)	0.00(0.00)	12.00(0.00)	0.00(0.00)	11.99(0.01)

*n* = 200, *K* = 3	TP	$\hat{s}$	TP	$\hat{s}$	TP	$\hat{s}$
IC_0_	1.00(0.00)	2.00(0.00)	0.97(0.02)	4.03(0.02)	0.98(0.01)	6.02(0.01)
IC_0.5_	1.00(0.00)	2.00(0.00)	0.97(0.02)	4.03(0.02)	0.98(0.01)	6.02(0.01)
IC_1_	1.00(0.00)	2.00(0.00)	0.97(0.02)	4.03(0.02)	0.98(0.01)	6.02(0.01)
BIC	0.00(0.00)	12.00(0.00)	0.00(0.00)	12.00(0.00)	0.00(0.00)	11.99(0.01)

**Table 5:** Simulation results for Setting IV, V and VI (standard errors in parenthesis)

	*s*_0_ = 2		*s*_0_ = 4		*s*_0_ = 6	
*n* = 50, *K* = 10	TP	$\hat{s}$	TP	$\hat{s}$	TP	$\hat{s}$
IC_0_	1.00(0.00)	2.00(0.00)	0.96(0.02)	3.98(0.02)	0.73(0.04)	5.83(0.06)
IC_0.5_	1.00(0.00)	2.00(0.00)	0.95(0.02)	3.97(0.02)	0.69(0.05)	5.77(0.06)
IC_1_	1.00(0.00)	2.00(0.00)	0.93(0.03)	3.93(0.03)	0.63(0.05)	5.57(0.07)
BIC	0.00(0.00)	11.83(0.05)	0.00(0.00)	11.82(0.04)	0.00(0.00)	11.86(0.04)

*n* = 50, *K* = 20	TP	$\hat{s}$	TP	$\hat{s}$	TP	$\hat{s}$
IC_0_	0.98(0.01)	2.02(0.01)	0.90(0.03)	4.10(0.03)	0.76(0.04)	6.06(0.05)
IC_0.5_	0.98(0.01)	2.02(0.01)	0.94(0.02)	3.98(0.02)	0.81(0.04)	5.99(0.04)
IC_1_	0.98(0.01)	2.02(0.01)	0.94(0.02)	3.94(0.02)	0.74(0.04)	5.81(0.05)
BIC	0.00(0.00)	12.00(0.00)	0.00(0.00)	12.00(0.00)	0.00(0.00)	11.99(0.01)

*n* = 50, *K* = 50	TP	$\hat{s}$	TP	$\hat{s}$	TP	$\hat{s}$
IC_0_	0.96(0.02)	2.04(0.02)	0.88(0.03)	4.12(0.03)	0.68(0.05)	6.57(0.13)
IC_0.5_	0.98(0.01)	2.02(0.01)	0.94(0.02)	4.04(0.02)	0.82(0.04)	6.06(0.04)
IC_1_	0.98(0.01)	2.02(0.01)	0.94(0.02)	4.02(0.02)	0.74(0.04)	5.75(0.05)
BIC	0.00(0.00)	12.00(0.00)	0.00(0.00)	12.00(0.00)	0.00(0.00)	12.00(0.00)

## Appendix A. More on the Identifiability Constraint

Let *π*(·) be a permutation function of {1,…,n}. Alternative to our estimator defined in Equation 3, one may consider

$$\left({\left\{{\hat{a}}_{i,\pi }^{\left(s\right)}\right\}}_{i},{\left\{{\hat{R}}_{k,\pi }^{\left(s\right)}\right\}}_{k}\right)=\underset{\underset{\mathrm{Vec}\left(R_{1}^{\left(s\right)}\right),\ldots ,\mathrm{Vec}\left(R_{K}^{\left(s\right)}\right)\in \Theta_{r}^{\left(s\right)}}{a_{1}^{\left(s\right)},\ldots ,a_{n}^{\left(s\right)}\in \Theta_{a}^{\left(s\right)}}}{\arg\max}l_{n}\left(Y;{\left\{a_{i}^{\left(s\right)}\right\}}_{i},{\left\{R_{k}^{\left(s\right)}\right\}}_{k}\right),$$

$$\;\text{subject to}\;\left[a_{\pi (1)}^{\left(s\right)},\ldots ,a_{\pi (s)}^{\left(s\right)}\right]=I_{s},$$

and the corresponding information criteria

$${\text{IC}}_{\pi }(s)=2l_{n}\left(Y;{\left\{{\hat{a}}_{i,\pi }^{\left(s\right)}\right\}}_{i},{\left\{{\hat{R}}_{k,\pi }^{\left(s\right)}\right\}}_{k}\right)-s\kappa (n,K).$$

Since $l_{n}\left(Y;{\left\{a_{i}^{\left(s\right)}\right\}}_{i},R_{k}^{\left(s\right)}{}_{k}\right)=l_{n}\left(Y;{\left\{C^{-1}a_{i}^{\left(s\right)}\right\}}_{i},{\left\{C^{T}R_{k}^{\left(s\right)}C\right\}}_{k}\right)$ for any invertible matrix $C\in {\mathbb{R}}^{s\times s},\left({\left\{{\hat{a}}_{i,\pi }^{\left(s\right)}\right\}}_{i},{\left\{{\hat{R}}_{k,\pi }^{\left(s\right)}\right\}}_{k}\right)$ is also the maximizer of $l_{n}\left(Y;{\left\{a_{i}^{\left(s\right)}\right\}}_{i},{\left\{R_{k}^{\left(s\right)}\right\}}_{k}\right)$ subject to the constraint that $\left[a_{\pi (1)}^{\left(s\right)},\ldots ,a_{\pi (s)}^{\left(s\right)}\right]$ is invertible. Similarly, the estimator $\left({\left\{{\hat{a}}_{i}^{\left(s\right)}\right\}}_{i},{\left\{{\hat{R}}_{k}^{\left(s\right)}\right\}}_{k}\right)$ is the maximizer of $l_{n}\left(Y;{\left\{a_{i}^{\left(s\right)}\right\}}_{i},{\left\{R_{k}^{\left(s\right)}\right\}}_{k}\right)$ subject to the constraint that $\left[a_{1}^{\left(s\right)},\ldots ,a_{s}^{\left(s\right)}\right]$ is invertible. As a result, we have IC(*s*) = IC_*π*_(*s*) as long as

(15)
$$\left[{\hat{a}}_{\pi (1)}^{\left(s\right)},\ldots ,{\hat{a}}_{\pi (s)}^{\left(s\right)}\right]\;\text{is invertible and}\;\left[{\hat{a}}_{1,\pi }^{\left(s\right)},\ldots ,{\hat{a}}_{s,\pi }^{\left(s\right)}\right]\;\text{is invertible}.$$

However, it remains unknown whether Equation 15 holds or not. Hence, there’s no guarantee that IC(*s*) = IC_*π*_(*s*). This means the choice of the identifiability constraint might affect the value of our proposed information criterion.

In the following, we prove

(16)
$$\mathrm{Pr}\left(\underset{\begin{array}{c} \pi \{1,\ldots ,n\}\to \{1,\ldots ,n\}\\ |\{\pi (1),\ldots ,\pi (n)\}|=n \end{array}}{\cap }\{\underset{s=1,\ldots ,s_{\max}}{\arg\max}\left\{{\mathrm{IC}}_{\pi (s)}\}=s_{0}\right\}\right)\to 1.$$

This means with probability tending to 1, all the information criteria with different identifiability constraints will select the true model. Therefore, the choice of the identifiability constraint will not affect the performance of our method. For any $s\in \left\{s_{0},\ldots ,s_{\max}\right\}$, let $A_{s,0,\pi }={\left[a_{\pi (1),0}^{\left(s\right)},\ldots ,a_{\pi (s),0}^{\left(s\right)}\right]}^{T}$,

$$a_{i,\pi }^{(s)*}={\left(A_{s,0,\pi }^{-1}\right)}^{T}a_{i,0}^{\left(s\right)}\;\text{and}\;R_{k,\pi }^{(s)*}=A_{s,0,\pi }R_{k,0}^{\left(s\right)}A_{s,0,\pi }^{T}.$$

We need the following condition.

(A4) Assume $a_{i,\pi }^{(s)*}\in \Theta_{a}^{\left(s\right)}$ and $\mathrm{vec}\left(R_{k,\pi }^{(s)*}\right)\in \Theta_{r}^{\left(s\right)},\;\forall i=1,\ldots ,n,\;k=1,\ldots ,K,\;s_{0}\leq s\leq s_{\max}$ and any permutation function *π*(·). In addition, assume $\sup_{x\in \Theta_{a}^{\left(s\right)}}\|x{\|}_{2}\leq \omega_{a}$, $\sup_{y\in \Theta_{r}^{\left(s\right)}}\|y{\|}_{2}\leq \omega_{r}$ for some $\omega_{a},\omega_{r}>0$.

### Corollary 1.

*Assume (A2)-(A4) hold,* $K\sim n^{l_{0}}$ *for some* $0\leq l_{0}\leq 1,\;s_{\max}=o\{\sqrt{n/\log(nK)}\}\lim\inf_{n}n^{\left(1-l_{0}\right)/2}\bar{K}\geq \exp\left(2\omega_{a}^{2}\omega_{r}\right)\sqrt{\log n}$. *Assume* κ(n,K) *satisfies Condition* (6). *Then,* (16) *is satisfied.*

## Appendix B. More on the Technical Conditions

### B.1. Discussion of Condition (A0)

In this section, we show the necessity of (A0) when the matrices $R_{1,0},\ldots ,R_{K,0}$ are symmetric. More specifically, when (A0) doesn’t hold, we show there exist some $0\leq s<s_{0},{\bar{a}}_{1,0},\ldots ,{\bar{a}}_{n,0}\in {\mathbb{R}}^{s}$ and ${\bar{R}}_{1,0},\ldots ,{\bar{R}}_{K,0}\in {\mathbb{R}}^{s\times s}$ such that

(17)
$${\bar{a}}_{i,0}^{T}{\bar{R}}_{k,0}{\bar{a}}_{j,0}=a_{i,0}^{T}R_{k,0}a_{j,0},\;\forall 1\leq i,j\leq n,1\leq k\leq K.$$

Let’s first consider the case where rank(***A***_0_) = *s* for some *s* < *s*_0_. Thus, it follows that

$$A_{0}={\bar{A}}_{0}C,$$

for some ${\bar{A}}_{0}\in {\mathbb{R}}^{n\times s}$ and $C\in {\mathbb{R}}^{s\times s_{0}}$. Set ${\bar{a}}_{i,0}$ to be the *i*th row of ${\bar{A}}_{0}$ and ${\bar{R}}_{k,0}=CR_{k,0}C^{T}$, Equation 17 is thus satisfied. In addition, the new matrix ${\bar{A}}_{0}$ shall have full column rank.

Let $R_{0}={\left(R_{1,0}^{T},\ldots ,R_{K,0}^{T}\right)}^{T},$. Consider the case where rank(***R***_0_) = *s* for some *s* < *s*_0_. It follows from the singular value decomposition that

(18)
$$R_{0}=U_{0}\Lambda_{0}V_{0}^{T},$$

for some diagonal matrix $\Lambda_{0}\in {\mathbb{R}}^{s\times s}$ and some matrices $U_{0}\in {\mathbb{R}}^{Ks_{0}\times s}$, $V_{0}\in {\mathbb{R}}^{s_{0}\times s}$ that satisfy $U_{0}^{T}U_{0}=V_{0}^{T}V_{0}=I_{s}$. Denoted by ***U***_*k*, 0_ the submatrix of ***U***_0_ formed by rows in $\left\{(k-1)s_{0}+1,(k-1)s_{0}+2,\cdots ,ks_{0}\right\}$ and columns in {1, 2,...,*s*}. It follows from Equation 18 that

$$R_{k,0}=U_{k,0}\Lambda_{0}V_{0}^{T},\;\forall 1\leq k\leq K.$$

Since ***R***_*k*,0_ is symmetric, we have $U_{k,0}\Lambda_{0}V_{0}^{T}=V_{0}\Lambda_{0}U_{k,0}^{T}=R_{k,0}$. Notice that $V_{0}^{T}V_{0}=I_{s}$. It follows that $U_{k,0}\Lambda_{0}=V_{0}\Lambda_{0}U_{k,0}^{T}V_{0}$. Therefore, we have

(19)
$$R_{k,0}=V_{0}\Lambda_{0}U_{k,0}^{T}V_{0}V_{0}^{T}.$$

Define ${\bar{a}}_{i,0}=V_{0}^{T}a_{i,0},\forall 1\leq i\leq n$ and ${\bar{R}}_{k,0}=\Lambda_{0}U_{k,0}^{T}V_{0}$. In view of Equation 19, it is immediate to see that Equation 17 holds. Since $V_{0}^{T}V_{0}=I_{s}$, we have ${\bar{R}}_{k,0}=V_{0}^{T}V_{0}\Lambda_{0}U_{k,0}^{T}V_{0}=V_{0}^{T}R_{k,0}V_{0}$. As a result, ${\bar{R}}_{1,0},\ldots ,{\bar{R}}_{K,0}$ are also symmetric. Suppose ${\bar{R}}_{0}={\left({\bar{R}}_{1,0}^{T},\cdots ,{\bar{R}}_{K,0}^{T}\right)}^{T}$ doesn’t have full column rank. Using the same arguments, we can find some $s^{\prime }<s,\;{\tilde{a}}_{1,0},\cdots ,{\tilde{a}}_{n,0}\in {\mathbb{R}}^{s^{\prime }},{\tilde{R}}_{1,0},\cdots ,{\tilde{R}}_{K,0}\in {\mathbb{R}}^{s^{\prime }\times s^{\prime }}$ such that

(20)
$${\tilde{a}}_{i,0}^{T}{\tilde{R}}_{k,0}{\tilde{a}}_{j,0}=a_{i,0}^{T}R_{k,0}a_{j,0},\;\forall 1\leq i,j\leq n,1\leq k\leq K.$$

We may repeat this procedure until we find some ${\tilde{a}}_{i,0}$, ${\tilde{R}}_{k,0}$’s satisfy Equation 20 and that the matrix ${\left({\tilde{R}}_{1,0}^{T},\cdots ,{\tilde{R}}_{K,0}^{T}\right)}^{T}$ has full column rank.

### B.2. Discussion of Condition (A1)

In this section, we consider an asymptotic framework where $a_{1,0},\ldots ,a_{n,0}$ are i.i.d according to some distribution function and show (A1) holds with probability tending to 1. For any *q*-dimensional vector q, let $\|q{\|}_{2}$ denote its Euclidean norm. For any *m* × *q* matrix ***Q***, $\|Q{\|}_{2}$ stands for the spectral norm of ***Q*** while $\|Q{\|}_{F}$ denotes its Frobenius norm. For simplicity, we assume $\Theta_{a}=\left\{x\in {\mathbb{R}}^{s_{0}}:\|x{\|}_{2}\leq \omega_{a}\right\}$ and $\Theta_{r}=\left\{y\in {\mathbb{R}}^{s_{0}^{2}}:\|y{\|}_{2}\leq \omega_{r}\right\}$. Assume $\max_{k=1,\ldots ,K}{\left\|R_{k,0}\right\|}_{F}=O(1),\;{\left\|a_{1,0}\right\|}_{2}$ is bounded with probability 1. In addition, assume there exist some constants $c_{0},\;t_{0}>0$ such that

(21)
$$\mathrm{Pr}\left({\left\|{\left[a_{1,0},\ldots ,a_{s_{0},0}\right]}^{-1}\right\|}_{F}>t\right)\leq c_{0}t^{-1},\;\forall t\leq t_{0}.$$

When *s*_0_ = 1, the above condition is closely related to the margin assumption (Tsybakov, 2004; Audibert and Tsybakov, 2007) in the classification literature. It automatically holds when *a*_1,0_ has a bounded probability density function.

In the following, we show with proper choice of *ω*_*a*_ and *ω*_*r*_, (A1) holds with probability tending to 1. By the identifiability constraints, ${\left\|a_{i}^{(s)*}\right\|}_{2}=1,\;\forall i=1,\ldots ,s$ and $s=s_{0},\ldots ,s_{\max}$. When $\omega_{a},\omega_{r}\to \infty$, we have for sufficiently large *n*,

(22)
$$\mathrm{Pr}\left(a_{i}^{(s)*}\in \Omega_{a}\right)=1,\;\forall 1\leq i\leq s,s_{0}\leq s\leq s_{\max}.$$

By the definition of ***A***_*s*,0_, we have

(23)
$$A_{s,0}^{-1}=\left(\begin{array}{cc} A_{s_{0},0}^{-1} & O_{s_{0}\times \left(s-s_{0}\right)}\\ -A_{\left(s_{0}+1\right):s,0}A_{s_{0},0}^{-1} & I_{s-s_{0}} \end{array}\right),$$

where $A_{\left(s_{0}+1\right):s,0}={\left[a_{s_{0}+1,0},\ldots ,a_{s,0}\right]}^{T}$ .It follows that

$$a_{i}^{(s)*}=\left(\begin{array}{c} A_{s_{0},0}^{-1}a_{i,0}\\ {\left(\underset{i-s_{0}-1}{\underset{︸}{0,\ldots ,0}},1,\underset{s-i}{\underset{︸}{0,\ldots ,0}}\right)}^{T}-A_{\left(s_{0}+1\right):s,0}A_{s_{0},0}^{-1}a_{i,0} \end{array}\right),\;\forall s<i\leq n,s_{0}\leq s\leq s_{\max}.$$

Under the given conditions, we have ${\left\|a_{i,0}\right\|}_{2}\leq \omega_{0}$ with probability 1 for some constant *ω*_0_ > 0. Therefore, we have

$$\underset{\underset{s<i\leq n}{s_{0}\leq s\leq s\leq s_{\max}}}{\max}{\left\|a_{i}^{{\left(s\right)}^{*}}\right\|}_{2}\leq \omega_{0}{\left\|A_{s_{0},0}^{-1}\right\|}_{2}+1+\sqrt{s_{\max}}\omega_{0}^{2}{\left\|A_{s_{0},0}^{-1}\right\|}_{2}\leq 1+(1+\sqrt{s_{\max}}\omega_{0})\omega_{0}{\left\|A_{s_{0},0}^{-1}\right\|}_{F}.$$

By (21), it is immediate to see that

$$\mathrm{Pr}\left(\underset{\underset{1\leq i\leq n}{s_{0}\leq s\leq s_{\max}}}{\max}{\left\|a_{i}^{{\left(s\right)}^{*}}\right\|}_{2}>\omega_{a}\right)\to 0,$$

as long as $\omega_{a}⩾\sqrt{s_{\text{max}}}$. This together with Equation 22 yields

$$\mathrm{Pr}\left(\underset{\underset{1\leq i\leq n}{{}_{s_{0}\leq s\leq s_{\max}}}}{\max}{\left\|a_{i}^{{\left(s\right)}^{*}}\right\|}_{2}>\omega_{a}\right)\to 0.$$

Combining Equation 23 with the definition of $R_{k}^{(s)*}$ yields

$$R_{k}^{(s)*}=\left(\begin{array}{cc} A_{s_{0},0}^{-1}R_{k,0}{\left(A_{s_{0},0}^{-1}\right)}^{T} & -A_{s_{0},0}^{-1}R_{k,0}{\left(A_{s_{0},0}^{-1}\right)}^{T}A_{(s_{0}+1):s,0}^{T}\\ -A_{\left(s_{0}+1\right):s,0}A_{s_{0},0}^{-1}R_{k,0}{\left(A_{s_{0},0}\right)}^{T} & A_{\left(s_{0}+1\right):s,0}A_{s_{0},0}^{-1}R_{k,0}{\left(A_{s_{0},0}^{-1}\right)}^{T}A_{\left(s_{0}+1\right);s,0}^{T} \end{array}\right)$$

Since $\max_{1\leq k\leq K}{\left\|R_{k,0}\right\|}_{F}=O(1),\;\max_{1\leq i\leq n}{\left\|a_{i,0}\right\|}_{2}=O(1)$ we have

$$\underset{1\leq k\leq K}{\max}{\left\|A_{s_{0},0}^{-1}R_{k,0}{\left(A_{s_{0},0}^{-1}\right)}^{T}\right\|}_{F}\leq \underset{1\leq k\leq K}{\max}{\left\|A_{s_{0},0}^{-1}\right\|}_{F}^{2}{\left\|R_{k,0}\right\|}_{F}=O\left({\left\|A_{s_{0},0}^{-1}\right\|}_{F}^{2}\right),$$

and

$$\;\underset{\underset{s_{0}\leq s\leq s_{\max}}{1\leq k\leq K}}{\max}{\left\|A_{\left(s_{0}+1\right):s,0}A_{s_{0},0}^{-1}R_{k,0}{\left(A_{s_{0},0}^{-1}\right)}^{T}\right\|}_{F}\;\leq \underset{\underset{s_{0}\leq s\leq \sin nx}{1\leq k\leq K}}{\max}{\left\|A_{s_{0},0}^{-1}\right\|}_{F}^{2}{\left\|A_{\left(s_{0}+1\right):s,0}\right\|}_{F}{\left\|R_{k,0}\right\|}_{F}=O\left(\sqrt{s_{\max}}{\left\|A_{s_{0},0}^{-1}\right\|}_{F}^{2}\right),\;\;\underset{\underset{s_{0}\leq s\leq s_{\max}}{1\leq k\leq K}}{\max}{\left\|A_{\left(s_{0}+1\right):s,0}A_{s_{0},0}^{-1}R_{k,0}{\left(A_{s_{0},0}^{-1}\right)}^{T}A_{\left(s_{0}+1\right):s,0}^{T}\right\|}_{F}\;\leq \underset{\underset{s_{0}\leq s\leq s_{\max}}{1\leq k\leq K}}{\max}{\left\|A_{s_{0},0}^{-1}\right\|}_{F}^{2}{\left\|A_{\left(s_{0}+1\right);s,0}\right\|}_{F}^{2}{\left\|R_{k,0}\right\|}_{F}=O\left(s_{\max}{\left\|A_{s_{0},0}^{-1}\right\|}_{F}^{2}\right).$$

By (21), we have $\max_{1\leq k\leq K,s_{0}\leq s\leq s_{\max}}{\left\|R_{k}^{(s)*}\right\|}_{F}\leq \omega_{r}$ with probability tending to 1, for any *ω*_*r*_ such that $\omega_{r}/s_{\max}\to \infty$.

### B.3. Discussion of Condition (A2)

Assume the matrix $\text{E}a_{1,0}a_{1,0}^{T}$ is positive definite. Since *s*_0_ is fixed, it follows from the law of large numbers that

$$\frac{1}{n}\sum_{i=1}^{n}a_{i,0}a_{i,0}^{T}\overset{P}{\to }{\text{E}a}_{1,0}a_{1,0}^{T}.$$

Therefore, (A2) holds with probability tending to 1.

## Appendix C. Proofs

In the following, we provide proofs of Lemma 1, Lemma 2 and Theorem 1. We define

$$l_{0}\left({\left\{a_{i}\right\}}_{i},{\left\{R_{k}\right\}}_{k}\right)=\text{E}l_{n}\left(Y;{\left\{a_{i}\right\}}_{i},{\left\{R_{k}\right\}}_{k}\right),$$

for any $a_{1},\ldots ,a_{n}\in {\mathbb{R}}^{s}$, $R_{1},\ldots ,R_{K}\in {\mathbb{R}}^{s\times s}$ and any integer *s* ≥ 1. Define $\theta_{ijk}={\left(a_{i}^{*}\right)}^{T}R_{k}a_{j}^{*}$ and ${\hat{\theta }}_{ijk}={\hat{a}}_{i}^{T}{\hat{R}}_{k}{\hat{a}}_{j}$

### C.1. Proof of Lemma 1

Assume there exist some ${\left\{a_{i}\right\}}_{i},{\left\{R_{k}\right\}}_{k}$ such that

$$\text{g}\left(a_{i,0}^{T}R_{k,0}a_{j,0}\right)=\text{g}\left(a_{i}^{T}R_{k}a_{j}\right),\;\forall i,j,k.$$

Since g(·) is strictly monotone, we have

$$a_{i,0}^{T}R_{k,0}a_{j,0}=a_{i}^{T}R_{k}a_{j},\;\forall i,j,k,$$

or equivalently,

$$\left(\begin{array}{c} AR_{1}\\ \vdots \\ AR_{K} \end{array}\right)A^{T}=\left(\begin{array}{c} A_{0}R_{1,0}\\ \vdots \\ A_{0}R_{K,0} \end{array}\right)A_{0}^{T}.$$

where $A={\left[a_{1},a_{2},\ldots ,a_{n}\right]}^{T}$. Thus, it follows that

$$\left(\begin{array}{c} A_{0}^{T}AR_{1}\\ \vdots \\ A_{0}^{T}AR_{K} \end{array}\right)A^{T}=\left(\begin{array}{c} A_{0}^{T}A_{0}R_{1,0}\\ \vdots \\ A_{0}^{T}A_{0}R_{K,0} \end{array}\right)A_{0}^{T}.$$

By (A0), the matrix $A_{0}^{T}A_{0}$ is invertible. As a result, we have

$$\left(\begin{array}{c} {\left(A_{0}^{T}A_{0}\right)}^{-1}A_{0}^{T}AR_{1}\\ \vdots \\ {\left(A_{0}^{T}A_{0}\right)}^{-1}A_{0}^{T}AR_{K} \end{array}\right)A^{T}=\left(\begin{array}{c} R_{1,0}\\ \vdots \\ R_{K,0} \end{array}\right)A_{0}^{T}.$$

Therefore,

$$\left(\sum_{k=1}^{K}R_{k,0}^{T}{\left(A_{0}^{T}A_{0}\right)}^{-1}A_{0}^{T}AR_{1}\right)A^{T}=\left(\sum_{k=1}^{K}R_{k,0}^{T}R_{k,0}\right)A_{0}^{T}.$$

Notice that the matrix $\sum_{k=1}^{K}R_{k,0}^{T}R_{k,0}$ is invertible under Condition (A0). It follows that

$$\underset{C}{\underset{︸}{{\left(\sum_{k=1}^{K}R_{k,0}^{T}R_{k,0}\right)}^{-1}\left(\sum_{k=1}^{K}R_{k,0}^{T}{\left(A_{0}^{T}A_{0}\right)}^{-1}A_{0}^{T}AR_{k}\right)}}A^{T}=A_{0}^{T}.$$

By Lemma 5.1 in Banerjee and Roy (2014), we have rank(***C***) ≥ rank(***A***_0_) = *s*_0_. Therefore, ***C*** is invertible. It follows that

(24)
$$A=A_{0}{\left(C^{T}\right)}^{-1},$$

or equivalently,

$$a_{i}=C^{-1}a_{i,0},\;\forall i=1,\ldots ,n.$$

By Equation 24, we obtain $A_{0}{\left(C^{T}\right)}^{-1}R_{k}C^{-1}A_{0}^{T}=A_{0}R_{k,0}A_{0}^{T},\forall k$ and hence

$$A_{0}^{T}A_{0}{\left(C^{T}\right)}^{-1}R_{k}C^{-1}A_{0}^{T}A_{0}=A_{0}^{T}A_{0}R_{k,0}A_{0}^{T}A_{0},\;\forall k=1,\ldots ,K.$$

Since $A_{0}^{T}A_{0}$ is invertible, this further implies ${\left(C^{T}\right)}^{-1}R_{k}C^{-1}=R_{k,0},\forall k,$ or equivalently,

$$R_{k}=C^{T}R_{k,0}C,\;\forall k=1,\ldots ,K.$$

The proof is hence completed.

### C.2. Proof of Lemma 2

To prove Lemma 2, we need the following lemma.

#### Lemma 4 (Mendelson et al. (2008), Lemma 2.3).

*Given d* ≥ 1, *and ε* > 0, *we have*

$$N\left(\varepsilon ,B_{2}^{d},\|\cdot {\|}_{2}\right)\leq {\left(1+\frac{2}{\varepsilon }\right)}^{d},$$

*where* $B_{2}^{d}$ *is the unit ball in* ${\mathbb{R}}^{d}$, *and* $N\left(\varepsilon ,\cdot ,\|\cdot {\|}_{2}\right)$ *the covering number with respect to the Euclidean metric (see Definition 2.2.3 in van der Vaart and Wellner (1996) for details)*.

Under Condition (Al), we have $l_{n}\left(Y;{\left\{a_{i}^{(s)*}\right\}}_{i},{\left\{R_{k}^{(s)*}\right\}}_{k}\right)\leq l_{n}\left(Y;{\left\{{\hat{a}}_{i}^{\left(s\right)}\right\}}_{i},{\left\{{\hat{R}}_{k}^{\left(s\right)}\right\}}_{k}\right).$ and hence

(25)
$$l_{n}\left(Y;{\left\{a_{i}^{(s)*}\right\}}_{i},{\left\{R_{k}^{(s)*}\right\}}_{k}\right)\leq l_{n}\left(Y;{\left\{{\hat{a}}_{i}^{\left(s\right)}\right\}}_{i},{\left\{{\hat{R}}_{k}^{\left(s\right)}\right\}}_{k}\right).$$

Besides, we have

(26)
$$\underset{i}{\max}{\left\|{\hat{a}}_{i}^{\left(s\right)}\right\|}_{2}\leq \omega_{a},\;\underset{k}{\max}{\left\|\mathrm{vec}\left({\hat{R}}_{k}^{\left(s\right)}\right)\right\|}_{2}\leq \omega_{r},\;\forall s\in \left\{s_{0},\ldots ,s_{\max}\right\},$$

and

(27)
$$\underset{i}{\max}{\left\|a_{i}^{*}\right\|}_{2}\leq \omega_{a},\;\underset{k}{\max}{\left\|\mathrm{vec}\left(R_{k}^{*}\right)\right\|}_{2}\leq \omega_{r}.$$

Therefore,

(28)
$$\;\underset{i,j,k}{\max}\left|{\left({\hat{a}}_{i}^{\left(s\right)}\right)}^{T}{\hat{R}}_{k}^{\left(s\right)}{\hat{a}}_{j}^{\left(s\right)}\right|\leq \left(\underset{i}{\max}{\left\|{\hat{a}}_{i}^{\left(s\right)}\right\|}_{2}^{2}\underset{k}{\max}{\left\|{\hat{R}}_{k}^{\left(s\right)}\right\|}_{2}\right)\leq \left(\underset{i}{\max}{\left\|{\hat{a}}_{i}^{\left(s\right)}\right\|}_{2}^{2}\underset{k}{\max}{\left\|{\hat{R}}_{k}^{\left(s\right)}\right\|}_{F}\right)\;\leq \left(\underset{{}_{i}}{\max}{\left\|{\hat{a}}_{i}^{\left(s\right)}\right\|}_{2}^{2}\underset{k}{\max}{\left\|\mathrm{vec}\left({\hat{R}}_{k}^{\left(s\right)}\right)\right\|}_{2}\right)\leq \omega_{a}^{2}\omega_{r},\;\forall s\in \left\{s_{0},\ldots ,s_{\max}\right\}.$$

Similarly, we can show

(29)
$$\underset{i,j,k}{\max}{\left\|{\left(a_{i}^{*}\right)}^{T}R_{k}^{*}a_{j}^{*}\right\|}_{2}\leq \omega_{a}^{2}\omega_{r}.$$

We define $\theta_{ijk}^{*}={\left(a_{i}^{*}\right)}^{T}R_{k}^{*}a_{j}^{*}\;\text{and}\;{\hat{\theta }}_{ijk}^{\left(s\right)}={\left({\hat{a}}_{i}^{\left(s\right)}\right)}^{T}{\hat{R}}_{k}^{\left(s\right)}{\hat{a}}_{j}^{\left(s\right)}$. It follows from a second-order Taylor expansion that

(30)
$$\text{g}\left(\theta_{ijk}^{*}\right)\left(\theta_{ijk}^{*}-{\hat{\theta }}_{ijk}^{\left(s\right)}\right)-\log\left\{1+\exp\left(\theta_{ijk}^{*}\right)\right\}+\log\left\{1+\exp\left({\hat{\theta }}_{ijk}^{\left(s\right)}\right)\right\}=\frac{\exp\left({\tilde{\theta }}_{ijk}^{\left(s\right)}\right){\left(\theta_{ijk}^{*}-{\hat{\theta }}_{ijk}^{\left(s\right)}\right)}^{2}}{2{\left\{1+\exp\left({\tilde{\theta }}_{ijk}^{\left(s\right)}\right)\right\}}^{2}},$$

for some ${\tilde{\theta }}_{ijk}^{\left(s\right)}$ lying on the line segment joining $\theta_{ijk}^{*}$ and ${\hat{\theta }}_{ijk}^{\left(s\right)}$. By (28) and (29), we have for any *i, j, k* and $s\in \left\{s_{0},\ldots ,s_{\max}\right\},\left|{\tilde{\theta }}_{ijk}^{\left(s\right)}\right|\leq \omega_{a}^{2}\omega_{r}$. This together with Equation 30 gives that

$$\;\text{g}\left(\theta_{ijk}^{*}\right)\theta_{ijk}^{*}-\log\left\{1+\exp\left(\theta_{ijk}^{*}\right)\right\}-\text{g}\left(\theta_{ijk}^{*}\right){\hat{\theta }}_{ijk}^{\left(s\right)}+\log\left\{1+\exp\left({\hat{\theta }}_{ijk}^{\left(s\right)}\right)\right\}\;\geq \frac{{\left(\theta_{ijk}^{*}-{\hat{\theta }}_{ijk}^{\left(s\right)}\right)}^{2}\exp\left(\omega_{a}^{2}\omega_{r}\right)}{2{\left\{1+\exp\left(\omega_{a}^{2}\omega_{r}\right)\right\}}^{2}}\geq \frac{{\left(\theta_{ijk}^{*}-{\hat{\theta }}_{ijk}^{\left(s\right)}\right)}^{2}\exp\left(\omega_{a}^{2}\omega_{r}\right)}{8\exp\left(2\omega_{a}^{2}\omega_{r}\right)}=\frac{{\left(\theta_{ijk}^{*}-{\hat{\theta }}_{ijk}^{\left(s\right)}\right)}^{2}}{8\exp\left(\omega_{a}^{2}\omega_{r}\right)},$$

and hence

(31)
$$\begin{array}{l} l_{0}\left({\left\{a_{i}^{*}\right\}}_{i},{\left\{R_{k}^{*}\right\}}_{k}\right)-l_{0}\left({\left\{{\hat{a}}_{i}^{\left(s\right)}\right\}}_{i},{\left\{{\hat{R}}_{k}^{\left(s\right)}\right\}}_{k}\right)\\ =\sum_{ijk}\left(\text{g}\left(\theta_{ijk}^{*}\right)\left(\theta_{ijk}^{*}-{\hat{\theta }}_{ijk}^{\left(s\right)}\right)-\log\frac{\left\{1+\exp\left(\theta_{ijk}^{*}\right)\right\}}{\left\{1+\exp\left({\hat{\theta }}_{ijk}^{\left(s\right)}\right)\right\}}\right)\geq \frac{\sum_{ijk}{\left(\theta_{ijk}^{*}-{\hat{\theta }}_{ijk}^{\left(s\right)}\right)}^{2}}{8\exp\left(\omega_{a}^{2}\omega_{r}\right)}. \end{array}$$

In the following, we provide an upper bound for

$$\begin{array}{l} \underset{{}_{s\in \left\{1,\ldots ,s_{\max}\right\}}}{\max}\underset{\underset{\underset{\max_{k}{\left\|\mathrm{vec}\left(R_{k}\right)\right\|}_{2}\leq \omega_{r}}{\max_{i}{\left\|a_{i}\right\|}_{2}\leq \omega_{a}}}{a_{i}\in {\mathbb{R}}^{a},R_{k}\in {\mathbb{R}}^{s\times s}}}{\sup}\left|l_{0}\left({\left\{a_{i}\right\}}_{i},{\left\{R_{k}\right\}}_{k}\right)-l_{n}\left(Y;{\left\{a_{i}\right\}}_{i},{\left\{R_{k}\right\}}_{k}\right)\right|\\ \underset{{}_{s\in \left\{1,\ldots ,s_{\max}\right\}}}{\max}\underset{\underset{\underset{\max_{k}{\left\|\mathrm{vec}\left(R_{k}\right)\right\|}_{2}\leq \omega_{r}}{\max_{i}{\left\|a_{i}\right\|}_{2}\leq \omega_{a}}}{a_{i}\in {\mathbb{R}}^{a},R_{k}\in {\mathbb{R}}^{s\times s}}}{\sup}\left|\sum_{ijk}\left(Y_{ijk}-\pi_{ijk}^{*}\right)a_{i}^{T}R_{k}a_{j}\right|, \end{array}$$

where $\pi_{ijk}^{*}=\exp\left(\theta_{ijk}^{*}\right)/\left\{1+\exp\left(\theta_{ijk}^{*}\right)\right\},\forall i,j,k$.

Let $\varepsilon_{a}=\omega_{a}/{\left(nK\right)}^{2}$ and $\left\{{\bar{a}}_{1}^{\left(s\right)},\ldots ,{\bar{a}}_{N_{s,\varepsilon_{a}}}^{\left(s\right)}\right\}$ be a minimal *ε*_*a*_-net of the vector space $\left(\left\{a\in {\mathbb{R}}^{s}:\|a{\|}_{2}\leq \omega_{a}\right\},\|\cdot {\|}_{2}\right)$. It follows from Lemma 4 that

(32)
$$N_{s,\varepsilon_{a}}=N\left(\varepsilon_{a},\left\{a\in {\mathbb{R}}^{s}:\|a{\|}_{2}\leq \omega_{a}\right\},\|\cdot {\|}_{2}\right)=N\left(1/{\left(nK\right)}^{2},B_{2}^{s},\|\cdot {\|}_{2}\right)\;\;\leq {\left(1+2n^{2}K^{2}\right)}^{s}\leq {\left(3nK\right)}^{2s}.$$

Let $\varepsilon_{r}=\omega_{r}/{\left(nK\right)}^{2}$, and $\left\{{\bar{R}}_{1}^{\left(s\right)},\ldots ,{\bar{R}}_{N_{s^{2},\varepsilon_{r}}}^{\left(s\right)}\right\}$ be a minimal *ε*_*r*_-net of the vector space $(\{R\in {\mathbb{R}}^{s\times s}:\|\mathrm{vec}(R){\|}_{2}\leq \omega \},\|\cdot {\|}_{F})$. For any *s* × *s* matrices ***Q***, we have $\|Q{\|}_{F}=\|\mathrm{vec}(Q){\|}_{2}$. Similar to (32), we can show that

(33)
$$N_{s^{2},\varepsilon_{r}}\leq {\left(3nK\right)}^{2s^{2}}.$$

Hence, for any $a_{i},a_{j}\in {\mathbb{R}}^{s}\;\text{and}\;R_{k}\in {\mathbb{R}}^{s\times s}$ satisfying ${\left\|a_{i}\right\|}_{2},{\left\|a_{j}\right\|}_{2}\leq \omega_{a},{\left\|\mathrm{vec}\left(R_{k}\right)\right\|}_{2}\leq \omega_{r}$ there exist some ${\bar{a}}_{l_{j}}^{\left(s\right)},{\bar{a}}_{l_{j}}^{\left(s\right)},{\bar{R}}_{t_{k}}^{\left(s\right)}$, such that

(34)
$${\left\|a_{i}-{\bar{a}}_{l_{i}}^{\left(s\right)}\right\|}_{2}\leq \frac{\omega_{a}}{{\left(nK\right)}^{2}},\;{\left\|a_{j}-{\bar{a}}_{l_{j}}^{\left(s\right)}\right\|}_{2}\leq \frac{\omega_{a}}{{\left(nK\right)}^{2}},\;{\left\|R_{k}-{\bar{R}}_{t_{k}}^{\left(s\right)}\right\|}_{F}\leq \frac{\omega_{r}}{{\left(nK\right)}^{2}}.$$

This further implies

(35)
$$\;\left|{\left(a_{i}\right)}^{T}R_{k}a_{j}-{\left({\bar{a}}_{l_{i}}^{\left(s\right)}\right)}^{T}{\bar{R}}_{t_{k}}^{\left(s\right)}{\bar{a}}_{l_{j}}^{\left(s\right)}\right|\leq \left|{\left(a_{i}\right)}^{T}R_{k}a_{j}-{\left({\bar{a}}_{l_{i}}^{\left(s\right)}\right)}^{T}R_{k}a_{j}\right|\;+\left|{\left({\bar{a}}_{l_{i}}^{\left(s\right)}\right)}^{T}R_{k}a_{j}-{\left({\bar{a}}_{l_{i}}^{\left(s\right)}\right)}^{T}{\bar{R}}_{t_{k}}^{\left(s\right)}a_{j}\right|+\left|{\left({\bar{a}}_{l_{i}}^{\left(s\right)}\right)}^{T}{\bar{R}}_{t_{k}}^{\left(s\right)}a_{j}-{\left({\bar{a}}_{l_{i}}^{\left(s\right)}\right)}^{T}{\bar{R}}_{t_{k}}^{\left(s\right)}{\bar{a}}_{l_{j}}^{\left(s\right)}\right|\;\leq {\left\|a_{i}-{\bar{a}}_{l_{i}}^{\left(s\right)}\right\|}_{2}{\left\|R_{k}\right\|}_{2}{\left\|a_{j}\right\|}_{2}+{\left\|{\bar{a}}_{l_{i}}^{\left(s\right)}\right\|}_{2}{\left\|R_{k}-{\bar{R}}_{t_{k}}^{\left(s\right)}\right\|}_{2}{\left\|a_{j}\right\|}_{2}+{\left\|{\bar{a}}_{i}^{\left(s\right)}\right\|}_{2}{\left\|{\bar{R}}_{t_{k}}^{\left(s\right)}\right\|}_{2}{\left\|a_{j}-{\bar{a}}_{l_{j}}^{\left(s\right)}\right\|}_{2}\;\leq {\left\|a_{i}-{\bar{a}}_{l_{i}}^{\left(s\right)}\right\|}_{2}{\left\|R_{k}\right\|}_{F}{\left\|a_{j}\right\|}_{2}+{\left\|{\bar{a}}_{l_{i}}^{\left(s\right)}\right\|}_{2}{\left\|R_{k}-{\bar{R}}_{t_{k}}^{\left(s\right)}\right\|}_{F}\left\|a_{j}\right\|+{\left\|{\bar{a}}_{i}^{\left(s\right)}\right\|}_{2}{\left\|{\bar{R}}_{t_{k}}^{\left(s\right)}\right\|}_{F}{\left\|a_{j}-{\bar{a}}_{l_{j}}^{\left(s\right)}\right\|}_{2}\;\leq 2\frac{\omega_{a}}{{\left(nK\right)}^{2}}\omega_{a}\omega_{r}+\frac{\omega_{r}}{{\left(nK\right)}^{2}}\omega_{a}^{2}=\frac{3\omega_{a}^{2}\omega_{r}}{{\left(nK\right)}^{2}}.$$

Therefore, we have

(36)
$$\underset{s\in \left\{1,\ldots ,s_{\max}\right\}}{\max}\frac{1}{s}\underset{\begin{array}{c} a_{i}\in {\mathbb{R}}^{s},R_{k}\in {\mathbb{R}}^{s\times s}\\ \max_{i}{\left\|a_{i}\right\|}_{2}\leq \omega_{a}\\ \max_{k}{\left\|\mathrm{vec}\left(R_{k}\right)\right\|}_{2}\leq \omega_{r} \end{array}}{\sup}\left|\sum_{ijk}\left(Y_{ijk}-\pi_{ijk}^{*}\right)a_{i}^{T}R_{k}a_{j}\right|\;\leq \underset{s\in \left\{1,\ldots ,s_{\max}\right\}}{\max}\frac{1}{s}\underset{\underset{t_{1},\ldots ,t_{K}\in \left\{1,\ldots ,N_{s^{2},\varepsilon_{r}}\right\}}{l_{1},\ldots ,l_{n}\in \left\{1,\ldots ,N_{s,\varepsilon_{a}}\right\}}}{\max}\left|\sum_{ijk}\left(Y_{ijk}-\pi_{ijk}^{*}\right){\left({\bar{a}}_{l_{i}}^{\left(s\right)}\right)}^{T}{\bar{R}}_{t_{k}}^{\left(s\right)}{\bar{a}}_{l_{j}}^{\left(s\right)}\right|+\sum_{ijk}\left|Y_{ijk}-\pi_{ijk}^{*}\right|\frac{3\omega_{a}^{2}\omega_{r}}{n^{2}K^{2}}\;\leq \underset{s\in \left\{1,\ldots ,s_{\max}\right\}}{\max}\frac{1}{s}\underset{\underset{t_{1},\ldots ,t_{K}\in \left\{1,\ldots ,N_{s^{2},\varepsilon_{r}}\right\}}{l_{1},\ldots ,l_{n}\in \left\{1,\ldots ,N_{s,\varepsilon_{a}}\right\}}}{\max}\left|\sum_{ijk}\left(Y_{ijk}-\pi_{ijk}^{*}\right){\left({\bar{a}}_{l_{i}}^{\left(s\right)}\right)}^{T}{\bar{R}}_{t_{k}}^{\left(s\right)}{\bar{a}}_{l_{j}}^{\left(s\right)}\right|+\frac{3\omega_{a}^{2}\omega_{r}}{K}.$$

By Bernstein’s inequality (van der Vaart and Wellner, 1996, Lemma 2.2.9), we obtain for any *t* > 0,

(37)
$$\begin{array}{cc} \underset{\underset{\underset{t_{1},\ldots ,t_{K}\in \left\{1,\ldots ,N_{s^{2},\varepsilon_{r}}\right\}}{{}_{l_{1},\ldots ,l_{n}\in \left\{1,\ldots ,N_{s,\varepsilon_{a}}\right\}}}}{s\in \left\{1,\ldots ,s_{\max}\right\}}}{\text{max}} & \mathrm{Pr}\left(\left|\sum_{ijk}\left(Y_{ijk}-\pi_{ijk}^{*}\right){\left({\bar{a}}_{l_{i}}^{\left(s\right)}\right)}^{T}{\bar{R}}_{t_{k}}^{\left(s\right)}{\bar{a}}_{l_{j}}^{\left(s\right)}\right|>t\right)\leq 2\exp\left(-\frac{t^{2}}{2\sigma_{0}^{2}+2M_{0}t/3}\right) \end{array},$$

where

$$M_{0}=\underset{s\in \left\{1,\ldots ,s_{\max}\right\}}{\max}\underset{t_{1},\ldots ,t_{K}\in \left\{1,\ldots ,N_{s^{2},\varepsilon_{r}}\right\}}{\underset{l_{1},\ldots ,l_{n}\in \left\{1,\ldots ,N_{s,\varepsilon_{a}}\right\}}{\max}}\underset{ijk}{\max}\left|Y_{ijk}-\pi_{ijk}^{*}\|{\left({\bar{a}}_{l_{i}}^{\left(s\right)}\right)}^{T}{\bar{R}}_{t_{k}}^{\left(s\right)}{\bar{a}}_{l_{j}}^{\left(s\right)}\right|,$$

$$\sigma_{0}^{2}=\underset{s\in \left\{1,\ldots ,s_{\max}\right\}}{\max}\underset{t_{1},\ldots ,t_{K}\in \left\{1,\ldots ,N_{s^{2},\varepsilon_{r}}\right\}}{\underset{l_{1},\ldots ,l_{n}\in \left\{1,\ldots ,N_{s,\varepsilon_{a}}\right\}}{\max}}\sum_{ijk}\text{E}{\left|Y_{ijk}-\pi_{ijk}^{*}\right|}^{2}{\left|{\left({\bar{a}}_{l_{i}}^{\left(s\right)}\right)}^{T}{\bar{R}}_{t_{k}}^{\left(s\right)}{\bar{a}}_{l_{j}}^{\left(s\right)}\right|}^{2}.$$

With some calculations, we can show that

(38)
$$\begin{array}{cccc} M_{0}\leq & \underset{\underset{t_{1},\ldots ,t_{K}\in \left\{1,\ldots ,N_{s^{2},\varepsilon_{r}}\right\}}{l_{1},\ldots ,l_{n}\in \left\{1,\ldots ,N_{s,\varepsilon_{a}}\right\}}}{\max} & {\left\|{\bar{a}}_{l_{i}}^{\left(s\right)}\right\|}_{2}{\left\|{\bar{R}}_{t_{k}}^{\left(s\right)}\right\|}_{2}{\left\|{\bar{a}}_{l_{j}}^{\left(s\right)}\right\|}_{2}\leq & \underset{\underset{t_{1},\ldots ,t_{K}\in \left\{1,\ldots ,N_{s^{2},\varepsilon_{r}}\right\}}{l_{1},\ldots ,l_{n}\in \left\{1,\ldots ,N_{s,\varepsilon_{a}}\right\}}}{\max}{\left\|{\bar{a}}_{l_{i}}^{\left(s\right)}\right\|}_{2}\left\|{\bar{R}}_{t_{k}}^{\left(s\right)}\right\|{}_{F}{\left\|{\bar{a}}_{l_{j}}^{\left(s\right)}\right\|}_{2} \end{array}\;\;\begin{array}{ccc} \leq & \underset{\underset{t_{1},\ldots ,t_{K}\in \left\{1,\ldots ,N_{s^{2},\varepsilon_{r}}\right\}}{l_{1},\ldots ,l_{n}\in \left\{1,\ldots ,N_{s,\varepsilon_{a}}\right\}}}{\max} & {\left\|{\bar{a}}_{l_{i}}^{\left(s\right)}\right\|}_{2}{\left\|\mathrm{vec}\left({\bar{R}}_{t_{k}}^{\left(s\right)}\right)\right\|}_{2}{\left\|{\bar{a}}_{l_{j}}^{\left(s\right)}\right\|}_{2}\leq \omega_{a}^{2}\omega_{r} \end{array},$$

and

(39)
$$\sigma_{0}^{2}\leq \underset{{}_{s\in \left\{1,\ldots ,s_{\max}\right\}}}{\max}\underset{\underset{t_{1},\ldots ,t_{K}\in \left\{1,\ldots ,N_{s^{2},\varepsilon_{r}}\right\}}{l_{1},\ldots ,l_{n}\in \left\{1,\ldots ,N_{s,\varepsilon_{a}}\right\}}}{\max}\sum_{ijk}\text{E}{\left|Y_{ijk}-\pi_{ijk}^{*}\right|}^{2}{\left\|{\bar{a}}_{l_{i}}^{\left(s\right)}\right\|}_{2}^{2}{\left\|\mathrm{vec}\left({\bar{R}}_{t_{k}}^{\left(s\right)}\right)\right\|}_{2}^{2}{\left\|{\bar{a}}_{l_{j}}^{\left(s\right)}\right\|}_{2}^{2}\;\;\leq \omega_{a}^{4}\omega_{r}^{2}\sum_{ijk}\text{E}{\left|Y_{ijk}-\pi_{ijk}^{*}\right|}^{2}\leq \omega_{a}^{4}\omega_{r}^{2}n^{2}K.$$

Let $t_{s}=5\omega_{a}^{2}\omega_{r}sn\sqrt{K\max(n,K)\log(nK)}$, we have

$$\;\underset{{}_{s\in \left\{1,\ldots ,s_{\max}\right\}}}{\max}\mathrm{Pr}\left(\underset{\underset{t_{1},\ldots ,t_{K}\in \left\{1,\ldots ,N_{s^{2},\varepsilon_{r}}\right\}}{l_{1},\ldots ,l_{n}\in \left\{1,\ldots ,N_{s,\varepsilon_{a}}\right\}}}{\max}\left|\sum_{ijk}\left(Y_{ijk}-\pi_{ijk}^{*}\right){\left({\bar{a}}_{l_{i}}^{\left(s\right)}\right)}^{T}{\bar{R}}_{t_{k}}^{\left(s\right)}{\bar{a}}_{l_{j}}^{\left(s\right)}\right|>t_{s}\right)$$

$$\leq \underset{\underset{t_{1},\ldots ,t_{K}\in \left\{1,\ldots ,N_{s^{2},\varepsilon_{r}}\right\}}{l_{1},\ldots ,l_{n}\in \left\{1,\ldots ,N_{s,\varepsilon_{a}}\right\}}}{\underset{s\in \left\{1,\ldots ,s_{\max}\right\}}{\text{max}}}N_{s,\varepsilon_{a}}^{n}N_{s^{2},\varepsilon_{r}}^{K}\mathrm{Pr}\left(\left|\sum_{ijk}\left(Y_{ijk}-\pi_{ijk}^{*}\right){\left({\bar{a}}_{l_{i}}^{\left(s\right)}\right)}^{T}{\bar{R}}_{t_{k}}^{\left(s\right)}{\bar{a}}_{l_{j}}^{\left(s\right)}\right|>t_{s}\right)$$

$$\leq \underset{\underset{t_{1},\ldots ,t_{K}\in \left\{1,\ldots ,N_{s^{2},\varepsilon_{r}}\right\}}{l_{1},\ldots ,l_{n}\in \left\{1,\ldots ,N_{s,\varepsilon_{a}}\right\}}}{\underset{s\in \left\{1,\ldots ,s_{\max}\right\}}{\text{max}}}{\left(3nK\right)}^{2sn+2s^{2}K}\mathrm{Pr}\left(\left|\sum_{ijk}\left(Y_{ijk}-\pi_{ijk}^{*}\right){\left({\bar{a}}_{l_{i}}^{\left(s\right)}\right)}^{T}{\bar{R}}_{t_{k}}^{\left(s\right)}{\bar{a}}_{l_{j}}^{\left(s\right)}\right|>t_{s}\right)$$

(40)
$$\begin{array}{l} \leq \underset{s\in \left\{1,\ldots ,s_{\max}\right\}}{\max}2\exp\left(-\frac{25\omega_{a}^{4}\omega_{r}^{2}s^{2}n^{2}K\max(n,K)\log(nK)}{2\omega_{a}^{4}\omega_{r}^{2}\{n^{2}K+sn\sqrt{K\max(n,K)\log(nK)}/3\}}+\left(2sn+2s^{2}K\right)\log(3nK)\right)\\ =\max_{s\in \left\{1,\ldots ,s_{\max}\right\}}2\exp\left(-\frac{25s^{2}\max(n,K)\log(nK)}{2+2s\sqrt{\max(n,K)\log(nK)}/(3n\sqrt{K})}+\left(2sn+2s^{2}K\right)\log(3nK)\right), \end{array}$$

where the first inequality is due to Bonferroni’s inequality, the second inequality follows by (32) and (33), the third inequality is due to (37)-(39).

Under the given conditions, we have $s\leq s_{\max}=o\{n/\log(nK)\}$ and hence

$$\frac{s\sqrt{\max(n,K)\log(nK)}}{n\sqrt{K}}\leq \frac{s\sqrt{n\log(nK)}}{n\sqrt{K}}+\frac{s\sqrt{K\log(nK)}}{n\sqrt{K}}\leq \frac{s_{\max}\sqrt{\log(nK)}}{\sqrt{n}}+\frac{s_{\max}\sqrt{\log(nK)}}{n}\ll 1.$$

It follows that for any $s\in \left\{1,\ldots ,s_{\max}\right\}$,

$$\begin{array}{r} \mathrm{Pr}\left(\underset{\underset{t_{1},\ldots ,t_{K}\in \left\{1,\ldots ,N_{s^{2},\varepsilon_{r}}\right\}}{l_{1},\ldots ,l_{n}\in \left\{1,\ldots ,N_{s,\varepsilon_{a}}\right\}}}{\max}\left|\sum_{ijk}{\left(Y_{ijk}-\pi_{ijk}^{\left(s\right)}\right)}^{T}{\left({\bar{a}}_{l_{j}}^{\left(s\right)}\right)}^{T}{\bar{R}}_{l_{k}}^{\left(s\right)}{\bar{a}}_{l_{j}}^{\left(s\right)}\right|>5\omega_{a}^{2}\omega_{r}sn\sqrt{K\max(n,K)\log(nK)}\right)\\ \leq 2\exp\left(-\frac{25s^{2}\max(n,K)\log(nK)}{2+1}+4s^{2}\max(n,K)\log(3nK)\right)\\ \leq 2\exp\left\{-4s^{2}\max(n,K)\log(nK)\right\}\leq 2\exp\left\{-4s^{2}n\log(nK)\right\}. \end{array}$$

By Bonferroni’s inequality, we have

$$\begin{array}{l} \mathrm{Pr}\left(\underset{s\in \left\{1,\ldots ,s_{\max}\right\}}{\cup }\{\underset{\underset{t_{1},\ldots ,t_{K}\in \left\{1,\ldots ,N_{s^{2},\varepsilon_{r}}\right\}}{l_{1},\ldots ,l_{n}\in \left\{1,\ldots ,N_{s,\varepsilon_{a}}\right\}}}{\max}\left|\sum_{ijk}\left(Y_{ijk}-\pi_{ijk}^{*}\right){\left({\bar{a}}_{l_{i}}^{\left(s\right)}\right)}^{T}{\bar{R}}_{t_{k}}^{\left(s\right)}{\bar{a}}_{l_{j}}^{\left(s\right)}\right|>t_{s}\}\right)\\ \leq \sum_{s=1}^{s_{\max}}2\exp\left\{-4s^{2}n\log(nK)\right\}\leq \sum_{s=1}^{+\infty }2\exp\{-4sn\log(nK)\}\leq \frac{2\exp\{-4n\log(nK)\}}{1-\exp\{-4n\log(nK)\}}\to 0. \end{array}$$

This together with (36) implies that

(41)
$$\underset{s\in \left\{1,\ldots ,s_{\max}\right\}}{\max}\frac{1}{s}\underset{\begin{array}{c} a_{i}\in {\mathbb{R}}^{s},R_{k}\in {\mathbb{R}}^{s\times s}\\ \max_{i}{\left\|a_{i}\right\|}_{2}\leq \omega_{a}\\ \max_{k}{\left\|\mathrm{vec}\left(R_{k}\right)\right\|}_{2}\leq \omega_{r} \end{array}}{\sup}\left|\sum_{ijk}\left(Y_{ijk}-\pi_{ijk}^{*}\right)a_{i}^{T}R_{k}a_{j}\right|\leq 6\omega_{a}^{2}\omega_{r}n\sqrt{K\max(n,K)\log(nK)},$$

with probability tending to 1. Combining this with (26) and (27), we obtain with probability tending to 1,

(42)
$$\max(\max_{s}\left|l_{0}\left({\left\{{\hat{a}}_{i}^{\left(s\right)}\right\}}_{i},{\left\{{\hat{R}}_{k}^{\left(s\right)}\right\}}_{k}\right)-l_{n}\left(Y;{\left\{{\hat{a}}_{i}^{\left(s\right)}\right\}}_{i},{\left\{{\hat{R}}_{k}^{\left(s\right)}\right\}}_{k}\right)\right|/s,$$

$$\left|l_{0}\left({\left\{a_{i}^{*}\right\}}_{i},{\left\{R_{k}^{*}\right\}}_{k}\right)-l_{n}\left(Y;{\left\{a_{i}^{*}\right\}}_{i},{\left\{R_{k}^{*}\right\}}_{k}\right)\right|/s_{0})\leq 6\omega_{a}^{2}\omega_{r}n\sqrt{K\max(n,K)\log(nK)}.$$

Therefore, it follows from (31) that

(43)
$$\underset{s\in \left\{s_{0},\ldots ,s_{\max}\right\}}{\max}\frac{1}{s}\sum_{ijk}{\left(\theta_{ijk}^{*}-{\hat{\theta }}_{ijk}^{\left(s\right)}\right)}^{2}$$

$$\leq \underset{s\in \left\{s_{0},\ldots ,s_{\max}\right\}}{\max}\frac{8\exp\left(\omega_{a}^{2}\omega_{r}\right)}{s}\left(l_{0}\left({\left\{a_{i}^{*}\right\}}_{i},{\left\{R_{k}^{*}\right\}}_{k}\right)-l_{0}\left({\left\{{\hat{a}}_{i}^{\left(s\right)}\right\}}_{i},{\left\{{\hat{R}}_{k}^{\left(s\right)}\right\}}_{k}\right)\right)$$

$$\leq \underset{s\in \left\{s_{0},\ldots ,s_{\max}\right\}}{\max}\frac{8\exp\left(\omega_{a}^{2}\omega_{r}\right)}{s}\left|l_{0}\left({\left\{{\hat{a}}_{i}^{\left(s\right)}\right\}}_{i},{\left\{{\hat{R}}_{k}^{\left(s\right)}\right\}}_{k}\right)-l_{n}\left(Y;{\left\{{\hat{a}}_{i}^{\left(s\right)}\right\}}_{i},{\left\{{\hat{R}}_{k}^{\left(s\right)}\right\}}_{k}\right)\right|$$

$$+\underset{s\in \left\{s_{0},\ldots ,s_{\max}\right\}}{\max}\frac{8\exp\left(\omega_{a}^{2}\omega_{r}\right)}{s}\left|l_{0}\left({\left\{a_{i}^{*}\right\}}_{i},{\left\{R_{k}^{*}\right\}}_{k}\right)-l_{n}\left(Y;{\left\{{\hat{a}}_{i}^{*}\right\}}_{i},{\left\{{\hat{R}}_{k}^{*}\right\}}_{k}\right)\right|$$

$$+\underset{s\in \left\{s_{0},\ldots ,s_{\max}\right\}}{\max}\frac{8\exp\left(\omega_{a}^{2}\omega_{r}\right)}{s}\left(l_{n}\left(Y;{\left\{a_{i}^{*}\right\}}_{i},{\left\{R_{k}^{*}\right\}}_{k}\right)-l_{n}\left(Y;{\left\{{\hat{a}}_{i}^{\left(s\right)}\right\}}_{i},{\left\{{\hat{R}}_{k}^{\left(s\right)}\right\}}_{k}\right)\right)$$

$$\leq \underset{s\in \left\{s_{0},\ldots ,s_{\max}\right\}}{\max}\frac{8\exp\left(\omega_{a}^{2}\omega_{r}\right)}{s}\left|l_{0}\left({\left\{{\hat{a}}_{i}^{\left(s\right)}\right\}}_{i},{\left\{{\hat{R}}_{k}^{\left(s\right)}\right\}}_{k}\right)-l_{n}\left(Y;{\left\{{\hat{a}}_{i}^{\left(s\right)}\right\}}_{i},{\left\{{\hat{R}}_{k}^{\left(s\right)}\right\}}_{k}\right)\right|$$

$$+\underset{s\in \left\{s_{0},\ldots ,s_{\max}\right\}}{\max}\frac{8\exp\left(\omega_{a}^{2}\omega_{r}\right)}{s}\left|l_{0}\left({\left\{a_{i}^{*}\right\}}_{i},{\left\{R_{k}^{*}\right\}}_{k}\right)-l_{n}\left(Y;{\left\{a_{i}^{*}\right\}}_{i},{\left\{R_{k}^{*}\right\}}_{k}\right)\right|$$

$$\leq 96\omega_{a}^{2}\omega_{r}\exp\left(\omega_{a}^{2}\omega_{r}\right)n\sqrt{K\max(n,K)\log(nK)},$$

where the third inequality is due to that $l_{n}\left(Y;{\left\{{\hat{a}}_{i}^{\left(s\right)}\right\}}_{i},{\left\{{\hat{R}}_{k}^{\left(s\right)}\right\}}_{k}\right)\geq l_{n}\left(Y;{\left\{a_{i}^{*}\right\}}_{i},{\left\{R_{k}^{*}\right\}}_{k}\right)$, for all $s\in \left\{s_{0},\ldots ,s_{\max}\right\}$.

Let $r_{n,K}={\left(n+K\right)}^{-1/2}{\left(\log n+\log K\right)}^{-1/2}$. As n→∞, we have

$$r_{n,K}^{2}n\sqrt{K\max(n,K)\log(nK)}\leq r_{n,K}^{2}n\sqrt{K(n+K)\log(nK)}=\frac{n\sqrt{K}}{\sqrt{\left(n+K)\log(nK\right)}}\ll \sqrt{nK}.$$

Since $\omega_{a}^{2}\omega_{r}\leq \exp\left(\omega_{a}^{2}\omega_{r}\right)$, it follows from (43) that

(44)
$$\mathrm{Pr}\left(\underset{s\in \left\{s_{0},\ldots ,s_{\max}\right\}}{\max}\frac{r_{n,K}^{2}}{s^{2}\exp\left(2\omega_{a}^{2}\omega_{r}\right)}\sum_{ijk}{\left(\theta_{ijk}^{*}-{\hat{\theta }}_{ijk}^{\left(s\right)}\right)}^{2}\geq \sqrt{nK}\right)\to 0.$$

For any integer *m* ≥ 1, define

$$S_{m}^{\left(s\right)}=\{\left({\left\{a_{i}^{\left(s\right)}\right\}}_{i},{\left\{R_{k}^{\left(s\right)}\right\}}_{k}\right):m-1<\frac{r_{n,K}}{s\exp\left(\omega_{a}^{2}\omega_{r}\right)}\sqrt{\sum_{ijk}{\left\{\theta_{ijk}^{*}-{\left(a_{i}^{\left(s\right)}\right)}^{T}R_{k}^{\left(s\right)}a_{j}^{\left(s\right)}\right\}}^{2}}\leq m,\;\;a_{i}^{\left(s\right)}\in {\mathbb{R}}^{s},\forall i,R_{k}^{\left(s\right)}\in {\mathbb{R}}^{s\times s},\forall k,\underset{i}{\max}{\left\|a_{i}^{\left(s\right)}\right\|}_{2}\leq \omega_{a},\underset{k}{\max}{\left\|\mathrm{vec}\left(R_{k}^{\left(s\right)}\right)\right\|}_{2}\leq \omega_{r}\}.$$

For any $\left({\left\{a_{i}^{\left(s\right)}\right\}}_{i},{\left\{R_{k}^{\left(s\right)}\right\}}_{k}\right)\in S_{m}^{\left(s\right)}$, similar to (31), we can show that

(45)
$$l_{0}\left({\left\{a_{i}^{*}\right\}}_{i},{\left\{R_{k}^{*}\right\}}_{k}\right)-l_{0}\left({\left\{a_{i}^{\left(s\right)}\right\}}_{i},{\left\{R_{k}^{\left(s\right)}\right\}}_{k}\right)\geq \frac{\sum_{ijk}{\left\{\theta_{ijk}^{*}-{\left(a_{i}^{\left(s\right)}\right)}^{T}R_{k}^{\left(s\right)}a_{j}^{\left(s\right)}\right\}}^{2}}{8\exp\left(\omega_{a}^{2}\omega_{r}\right)}\geq \frac{{\left(m-1\right)}^{2}s^{2}r_{n,K}^{-2}}{8\exp\left(-\omega_{a}^{2}\omega_{r}\right)}.$$

The event $\left({\left\{{\hat{a}}_{i}^{\left(s\right)}\right\}}_{i},{\left\{{\hat{R}}_{k}^{\left(s\right)}\right\}}_{k}\right)\in S_{m}^{\left(s\right)}$ implies that

$$\underset{\left({\left\{a_{i}^{\left(s\right)}\right\}}_{i},{\left\{R_{k}^{\left(s\right)}\right\}}_{k}\right)\in S_{m}^{\left(s\right)}}{\sup}l_{n}\left(Y;{\left\{a_{i}^{\left(s\right)}\right\}}_{i},{\left\{R_{k}^{\left(s\right)}\right\}}_{k}\right)\geq l_{n}\left(Y;{\left\{a_{i}^{*}\right\}}_{i},{\left\{R_{k}^{*}\right\}}_{k}\right).$$

It follows from (45) that

(46)
$$\underset{\left({\left\{a_{i}^{\left(s\right)}\right\}}_{i},{\left\{R_{k}^{\left(s\right)}\right\}}_{k}\right)\in S_{m}^{\left(s\right)}}{\sup}\left|\sum_{ijk}\left(Y_{ijk}-\pi_{ijk}^{*}\right)\left\{\theta_{ijk}^{*}-{\left(a_{i}^{\left(s\right)}\right)}^{T}R_{k}^{\left(s\right)}a_{j}^{\left(s\right)}\right\}\right|\geq \frac{{\left(m-1\right)}^{2}s^{2}\exp\left(\omega_{a}^{2}\omega_{r}\right)}{8r_{n,K}^{2}}.$$

For any {*l*_*i*_}_*i*_ and {*t*_*k*_}_*k*_ satisfying (34), it follows from (35) that

$$\sum_{ijk}{\left\{\theta_{ijk}^{*}-{\left({\bar{a}}_{l_{i}}^{\left(s\right)}\right)}^{T}{\bar{R}}_{t_{k}}^{\left(s\right)}{\bar{a}}_{l_{j}}^{\left(s\right)}\right\}}^{2}$$

$$\leq \sum_{ijk}{\left\{\theta_{ijk}^{*}-{\left(a_{i}^{\left(s\right)}\right)}^{T}R_{k}^{\left(s\right)}a_{j}^{\left(s\right)}+{\left(a_{i}^{\left(s\right)}\right)}^{T}R_{k}^{\left(s\right)}a_{j}^{\left(s\right)}-{\left({\bar{a}}_{l_{i}}^{\left(s\right)}\right)}^{T}{\bar{R}}_{t_{k}}^{\left(s\right)}{\bar{a}}_{l_{j}}^{\left(s\right)}\right\}}^{2}$$

(47)
$$\begin{array}{l} \leq 2\sum_{ijk}\left({\left\{\theta_{ijk}^{*}-{\left(a_{i}^{\left(s\right)}\right)}^{T}R_{k}^{\left(s\right)}a_{j}^{\left(s\right)}\right\}}^{2}+{\left\{{\left(a_{i}^{\left(s\right)}\right)}^{T}R_{k}^{\left(s\right)}a_{j}^{\left(s\right)}-{\left({\bar{a}}_{l_{i}}^{\left(s\right)}\right)}^{T}{\bar{R}}_{t_{k}}^{\left(s\right)}{\bar{a}}_{l_{j}}^{\left(s\right)}\right\}}^{2}\right)\\ \leq \frac{2m^{2}s^{2}\exp\left(2\omega_{a}^{2}\omega_{r}\right)}{r_{n,K}^{2}}+\frac{6\omega_{a}^{2}\omega_{r}}{K}\leq \frac{3m^{2}s^{2}\exp\left(2\omega_{a}^{2}\omega_{r}\right)}{r_{n,K}^{2}}. \end{array}$$

Let $\Lambda_{m}^{\left(s\right)}=\left\{\left({\left\{l_{i}\right\}}_{i},{\left\{t_{k}\right\}}_{k}\right):\sum_{ijk}{\left\{\theta_{ijk}^{*}-{\left({\bar{a}}_{l_{i}}^{\left(s\right)}\right)}^{T}{\bar{R}}_{t_{k}}^{\left(s\right)}{\bar{a}}_{l_{j}}^{\left(s\right)}\right\}}^{2}\leq 3m^{2}s^{2}\exp\left(2\omega_{a}^{2}\omega_{r}\right)/r_{n,K}^{2}\right\}$. Similar to (36), we can show

$$\underset{s\in \left\{s_{0},\ldots ,s_{\max}\right\}}{\max}\frac{1}{s^{2}}\underset{\left({\left\{a_{i}^{\left(s\right)}\right\}}_{i},{\left\{R_{k}^{\left(s\right)}\right\}}_{k}\right)\in S_{m}^{\left(s\right)}}{\sup}\left|\sum_{ijk}\left(Y_{ijk}-\pi_{ijk}^{*}\right)\left\{\theta_{ijk}^{*}-{\left(a_{i}^{\left(s\right)}\right)}^{T}R_{k}^{\left(s\right)}a_{j}^{\left(s\right)}\right\}\right|\;\leq \underset{s\in \left\{s_{0},\ldots ,s_{\max}\right\}}{\max}\frac{1}{s^{2}}\underset{\begin{array}{c} \begin{array}{l} l_{1},\ldots ,l_{n}\in \left\{1,\ldots ,N_{s},\varepsilon_{a}\right\}\\ t_{1},\ldots ,t_{K}\in \left\{1,\ldots ,N_{s^{2},\varepsilon_{r}}\right\} \end{array}\\ \left({\left\{1_{i}\right\}}_{i},{\left\{t_{k}\right\}}_{k}\right)\in \Lambda_{m}^{\left(s\right)} \end{array}}{\max}\left|\sum_{ijk}\left(Y_{ijk}-\pi_{ijk}^{*}\right)\left\{{\left({\bar{a}}_{l_{i}}^{\left(s\right)}\right)}^{T}{\bar{R}}_{t_{k}}^{\left(s\right)}{\bar{a}}_{l_{j}}^{\left(s\right)}-\theta_{ijk}^{*}\right\}\right|+\frac{3\omega_{a}^{2}\omega_{r}}{K}.$$

Under the event defined in (46), for any *m* ≥ 9, we have

$$\underset{s\in \left\{s_{0},\ldots ,s_{\max}\right\}}{\max}\frac{1}{s^{2}}\underset{\begin{array}{c} \begin{array}{l} l_{1},\ldots ,l_{n}\in \left\{1,\ldots ,N_{s},\varepsilon_{a}\right\}\\ t_{1},\ldots ,t_{K}\in \left\{1,\ldots ,N_{s^{2},\varepsilon_{r}}\right\} \end{array}\\ \left({\left\{1_{i}\right\}}_{i},{\left\{t_{k}\right\}}_{k}\right)\in \Lambda_{m}^{\left(s\right)} \end{array}}{\max}\left|\sum_{ijk}\left(Y_{ijk}-\pi_{ijk}^{*}\right)\left\{{\left({\bar{a}}_{l_{i}}^{\left(s\right)}\right)}^{T}{\bar{R}}_{t_{k}}^{\left(s\right)}{\bar{a}}_{l_{j}}^{\left(s\right)}-\theta_{ijk}^{*}\right\}\right|$$

$$\geq \frac{{\left(m-1\right)}^{2}\exp(\omega_{a}^{2}\omega_{r})}{8r_{n,K}^{2}}-\frac{3\omega_{a}^{2}\omega_{r}}{K}\geq \frac{{\left(m-1\right)}^{2}\exp(\omega_{a}^{2}\omega_{r})}{16r_{n,K}^{2}}+\frac{4\exp(\omega_{a}^{2}\omega_{r})}{r_{n,K}^{2}}-\frac{3\omega_{a}^{2}\omega_{r}}{K}\geq \frac{{\left(m-1\right)}^{2}\exp(\omega_{a}^{2}\omega_{r})}{16r_{n,K}^{2}}.$$

Define

$${\left(\sigma_{m}^{\left(s\right)}\right)}^{2}=\underset{\begin{array}{c} \begin{array}{l} l_{1},\ldots ,l_{n}\in \left\{1,\ldots ,N_{s,\varepsilon_{n,K}}\right\}\\ t_{1},\ldots ,t_{K}\in \left\{1,\ldots ,N_{s^{2},\varepsilon_{n,K}}\right\} \end{array}\\ \left({\left\{1_{i}\right\}}_{i},{\left\{t_{k}\right\}}_{k}\right)\in \Lambda_{m}^{\left(s\right)} \end{array}}{\max}\sum_{ijk}\text{E}{\left|Y_{ijk}-\pi_{ijk}^{*}\right|}^{2}{\left\{{\left({\bar{a}}_{l_{i}}^{\left(s\right)}\right)}^{T}{\bar{R}}_{t_{k}}^{\left(s\right)}{\bar{a}}_{l_{j}}^{\left(s\right)}-\theta_{ijk}^{*}\right\}}^{2}.$$

By (47), it is immediate to see that

(48)
$${\left(\sigma_{m}^{\left(s\right)}\right)}^{2}\leq 3m^{2}s^{2}\exp\left(2\omega_{a}^{2}\omega_{r}\right)/r_{n,K}^{2}.$$

Similar to (37) and (40), we can show there exist some constants *J*_0_ > 0, *K*_0_ > 0 such that for any *m* ≥ *J*_0_ and any *s* such that *s*_0_ ≤ *s* ≤ *s*_max_,

$$\;\mathrm{Pr}\left(\frac{1}{s^{2}}\underset{\begin{array}{c} \begin{array}{l} l_{1},\ldots ,l_{n}\in \left\{1,\ldots ,N_{s,\varepsilon_{a}}\right\}\\ t_{1},\ldots ,t_{K}\in \left\{1,\ldots ,N_{s^{2},\varepsilon_{r}}\right\} \end{array}\\ \left({\left\{1_{i}\right\}}_{i},{\left\{t_{k}\right\}}_{k}\right)\in \Lambda_{m}^{\left(s\right)} \end{array}}{\max}\left|\sum_{ijk}\left(Y_{ijk}-\pi_{ijk}^{*}\right)\left\{{\left({\bar{a}}_{l_{i}}^{\left(s\right)}\right)}^{T}{\bar{R}}_{t_{k}}^{\left(s\right)}{\bar{a}}_{l_{j}}^{\left(s\right)}-\theta_{ijk}^{*}\right\}\right|\geq \frac{{\left(m-1\right)}^{2}\exp\left(\omega_{a}^{2}\omega_{r}\right)}{16r_{n,K}^{2}}\right)$$

$$\leq 2\exp\left(-\frac{{\left(m-1\right)}^{4}s^{4}\exp\left(2\omega_{a}^{2}\omega_{r}\right)/\left(256r_{n,K}^{4}\right)}{2{\left(\sigma_{m}^{\left(s\right)}\right)}^{2}+{\left(m-1\right)}^{2}s^{2}\omega_{a}^{2}\omega_{r}\exp\left(\omega_{a}\omega_{r}\right)M_{0}/\left(24r_{n,K}^{2}\right)}+\left(2sn+2s^{2}K\right)\log(3nK)\right)$$

$$\leq 2\exp\left(-\frac{{\left(m-1\right)}^{4}s^{4}/\left(256r_{n,K}^{4}\right)}{6m^{2}s^{2}/r_{n,K}^{2}+{\left(m-1\right)}^{2}s^{2}/\left(24r_{n,K}^{2}\right)}+\left(2sn+2s^{2}K\right)\log(3nK)\right)\leq 2\exp\left(-K_{0}m^{2}s^{2}r_{n,K}^{-2}\right),$$

where the second inequality is due to (38), (48) and the fact that $\omega_{a}^{2}\omega_{r}\leq \exp\left(\omega_{a}^{2}\omega_{r}\right).$ This yields

(49)
$$\mathrm{Pr}\left(\left({\left\{{\hat{a}}_{i}^{\left(s\right)}\right\}}_{i},{\left\{{\hat{R}}_{k}^{\left(s\right)}\right\}}_{k}\right)\in S_{m}^{\left(s\right)}\right)\leq 2\exp\left(-K_{0}m^{2}s^{2}r_{n,K}^{-2}\right).$$

Let *J*_*n*,*K*_ be the integer such that $J_{n,K}-1\leq {\left(nK\right)}^{1/4}\leq J_{n,K}.$ In view of (44), we have for any *m* ≥ *J*_0_,

$$\begin{array}{l} \mathrm{Pr}\left(\underset{s\in \left\{s_{0},\ldots ,s_{\max}\right\}}{\max}\frac{r_{n,K}}{s\exp\left(\omega_{a}^{2}\omega_{r}\right)}\sqrt{\sum_{ijk}{\left(\theta_{ijk}^{*}-{\hat{\theta }}_{ijk}^{\left(s\right)}\right)}^{2}}>m\right)\leq \sum_{s=s_{0}}^{s_{\max}}\sum_{l=m}^{J_{n,K}}2\exp\left(-K_{0}l^{2}s^{2}r_{n,K}^{-2}\right)+o(1)\\ \;\leq \sum_{s=1}^{+\infty }\sum_{l=1}^{+\infty }2\exp\left(-K_{0}l{\mathrm{mss}}_{0}r_{n,K}^{-2}\right)+o(1)\leq \frac{2\exp\left(-s_{0}mK_{0}r_{n,K}^{-2}\right)}{1-2\exp\left(-s_{0}mK_{0}r_{n,K}^{-2}\right)}+o(1)=o(1). \end{array}$$

This implies there exists some constant *C*_0_ > 0 such that the following event occurs with probability tending to 1,

(50)
$$\underset{s\in \left\{s_{0},\ldots ,s_{\max}\right\}}{\max}\frac{1}{s^{2}}\sum_{ijk}{\left({\hat{\theta }}_{ijk}^{\left(s\right)}-\theta_{ijk}^{*}\right)}^{2}\leq C_{0}\exp\left(\omega_{a}^{2}\omega_{r}\right)r_{n,K}^{-2},$$

The proof is hence completed.

### C.3. Proof of Lemma 3

For any *s* < *s*_0_, we have

(51)
$$\;\sum_{ijk}{\left({\left({\hat{a}}_{i}^{\left(s\right)}\right)}^{T}{\hat{R}}_{k}^{\left(s\right)}{\hat{a}}_{j}^{\left(s\right)}-{\left(a_{i}^{*}\right)}^{T}R_{k}^{*}a_{j}^{*}\right)}^{2}\;\geq \underset{\begin{array}{l} a_{1}^{\left(s\right)},\ldots ,a_{n}^{\left(s\right)}\in {\mathbb{R}}^{s}\\ R_{1}^{\left(s\right)},\ldots ,R_{K}^{\left(s\right)}\in {\mathbb{R}}^{s\times s} \end{array}}{\inf}\sum_{ijk}{\left({\left(a_{i}^{\left(s\right)}\right)}^{T}R_{k}^{\left(s\right)}a_{j}^{\left(s\right)}-{\left(a_{i}^{*}\right)}^{T}R_{k}^{*}a_{j}^{*}\right)}^{2}$$

$$=\underset{\begin{array}{c} A^{\left(s\right)}\in {\mathbb{R}}^{n\times s}\\ R_{1}^{\left(s\right)},\ldots ,R_{K}^{\left(s\right)}\in {\mathbb{R}}^{s\times s} \end{array}}{\inf}\sum_{k=1}^{K}{\left\|A^{\left(s\right)}R_{k}^{\left(s\right)}{\left(A^{\left(s\right)}\right)}^{T}-A_{0}R_{k,0}A_{0}^{T}\right\|}_{F}^{2}$$

$$=\underset{\begin{array}{c} A^{\left(s\right)}\in {\mathbb{R}}^{n\times s}\\ R_{1}^{\left(s\right)},\ldots ,R_{K}^{\left(s\right)}\in {\mathbb{R}}^{s\times s} \end{array}}{\inf}{\left\|\left(\begin{array}{c} A^{\left(s\right)}R_{1}^{\left(s\right)}\\ \vdots \\ A^{\left(s\right)}R_{K}^{\left(s\right)} \end{array}\right){\left(A^{\left(s\right)}\right)}^{T}-\underset{B_{0}}{\underset{︸}{\left(\begin{array}{c} A_{0}R_{1,0}\\ \vdots \\ A_{0}R_{K,0} \end{array}\right)}}A_{0}^{T}\right\|}_{F}^{2}$$

$$\geq \underset{A^{\left(s\right)}\in {\mathbb{R}}^{n\times s},B^{\left(s\right)}\in {\mathbb{R}}^{nK\times s}}{\inf}{\left\|B^{\left(s\right)}{\left(A^{\left(s\right)}\right)}^{T}-B_{0}A_{0}^{T}\right\|}_{F}^{2}.$$

Define

$$\left({\hat{A}}^{\left(s\right)},{\hat{B}}^{\left(s\right)}\right)=\arg\underset{\begin{array}{c} A^{\left(s\right)}\in {\mathbb{R}}^{n\times s}\\ B^{\left(s\right)}\in {\mathbb{R}}^{nK\times s} \end{array}}{\min}{\left\|B^{\left(s\right)}{\left(A^{\left(s\right)}\right)}^{T}-B_{0}A_{0}^{T}\right\|}_{F}^{2}.$$

The above minimizers are not unique. Notice that rank $\left(B_{0}A_{0}^{T}\right)\leq \mathrm{rank}\left(A_{0}^{T}\right)\leq s_{0}.$. Assume $B_{0}A_{0}^{T}$ has the following singular value decomposition,

$$B_{0}A_{0}^{T}=nU_{n}\Lambda_{n}V_{n}^{T},$$

for some $U_{n}\in {\mathbb{R}}^{nK\times s_{0}},V_{n}\in {\mathbb{R}}^{n\times s_{0}}\;\text{such that}\;U_{n}^{T}U_{n}=V_{n}^{T}V_{n}=I_{s_{0}},$ and some diagonal matrix

$$\Lambda_{n}=\mathrm{diag}\left({\text{λ}}_{n}^{\left(1\right)},{\text{λ}}_{n}^{\left(2\right)},\ldots ,{\text{λ}}_{n}^{\left(s_{0}\right)}\right)$$

such that $\left|{\text{λ}}_{n}^{\left(1\right)}\right|\geq \left|{\text{λ}}_{n}^{\left(2\right)}\right|\geq \cdots \geq \left|{\text{λ}}_{n}^{\left(s_{0}\right)}\right|.$ Then one solution is given by

$${\hat{A}}^{\left(s\right)}=nV_{n}\Lambda_{n}U_{n}^{T}U_{n}^{\left(s\right)},\;{\hat{B}}^{\left(s\right)}=U_{n}^{\left(s\right)},$$

where $U_{n}^{\left(s\right)}$ is the submatrix of ***U***_*n*_ formed by its first *s* columns.

Since $U_{n}^{T}U_{n}=I_{s_{0}},$ we have

$${\hat{B}}^{\left(s\right)}{\left({\hat{A}}^{\left(s\right)}\right)}^{T}=nU_{n}^{\left(s\right)}{\left(U_{n}^{\left(s\right)}\right)}^{T}U_{n}\Lambda_{n}V_{n}^{T}=nU_{n}^{\left(s\right)}\left(I_{s},O_{s,s_{0}-s}\right)\Lambda_{n}V_{n}^{T}\;\;=nU_{n}\left(\begin{array}{cc} I_{s} & O_{s,s_{0}-s}\\ O_{s_{0}-s,s} & O_{s_{0}-s,s_{0}-s} \end{array}\right)\Lambda_{n}V_{n}^{T},$$

and hence

$${\hat{B}}^{\left(s\right)}{\left({\hat{A}}^{\left(s\right)}\right)}^{T}-B_{0}A_{0}^{T}=nU_{n}\left(\begin{array}{cc} I_{s} & O_{s,s_{0}-s}\\ O_{s_{0}-s,s} & O_{s_{0}-s,s_{0}-s} \end{array}\right)\Lambda_{n}V_{n}^{T}-nU_{n}I_{s_{0}}\Lambda_{n}V_{n}^{T}\;\;=-nU_{n}\left(\begin{array}{cc} O_{s} & O_{s,s0-s}\\ O_{s_{0}-s,s} & I_{s_{0}-s,s_{0}-s} \end{array}\right)\Lambda_{n}V_{n}^{T}.$$

This together with (51) implies that

(52)
$$\begin{array}{l} \sum_{ijk}{\left({\left({\hat{a}}_{i}^{\left(s\right)}\right)}^{T}{\hat{R}}_{k}^{\left(s\right)}{\hat{a}}_{j}^{\left(s\right)}-{\left(a_{i}^{*}\right)}^{T}R_{k}^{*}a_{j}^{*}\right)}^{2}\geq {\left\|{\hat{B}}^{\left(s\right)}{\left({\hat{A}}^{\left(s\right)}\right)}^{T}-B_{0}A_{0}^{T}\right\|}_{F}^{2}\\ \geq n^{2}\mathrm{trace}\left(U_{n}\left(\begin{array}{cc} O_{s} & O_{s,s_{0}-s}\\ O_{s_{0}-s,s} & I_{s_{0}-s,s_{0}-s} \end{array}\right)\Lambda_{n}V_{n}^{T}V_{n}\Lambda_{n}\left(\begin{array}{cc} O_{s} & O_{s,s_{0}-s}\\ O_{s_{0}-s,s} & I_{s_{0}-s,s_{0}-s} \end{array}\right)U_{n}^{T}\right)\\ =n^{2}\mathrm{trace}\left(U_{n}\left(\begin{array}{cc} O_{s} & O_{s,s_{0}-s}\\ O_{s_{0}-s,s} & I_{s_{0}-s,s_{0}-s} \end{array}\right)\Lambda_{n}^{2}\left(\begin{array}{cc} O_{s} & O_{s,s_{0}-s}\\ O_{s_{0}-s,s} & I_{s_{0}-s,s_{0}-s} \end{array}\right)U_{n}^{T}\right) \end{array}$$

(53)
$$\begin{array}{l} =n^{2}\mathrm{trace}\left(\Lambda_{n}\left(\begin{array}{cc} O_{s} & O_{s,s_{0}-s}\\ O_{s_{0}-s,s} & I_{s_{0}-s,s_{0}-s} \end{array}\right)U_{n}^{T}U_{n}\left(\begin{array}{cc} O_{s} & O_{s,s_{0}-s}\\ O_{s_{0}-s,s} & I_{s_{0}-s,s_{0}-s} \end{array}\right)\Lambda_{n}\right)\\ =n^{2}\mathrm{trace}\left(\Lambda_{n}\left(\begin{array}{cc} O_{s} & O_{s,s_{0}-s}\\ O_{s_{0}-s,s} & I_{s_{0}-s,s_{0}-s} \end{array}\right)\Lambda_{n}\right)\geq n^{2}{\left({\text{λ}}_{n}^{\left(s_{0}\right)}\right)}^{2}, \end{array}$$

where (52) is due to that $V_{n}^{T}V_{n}=I_{s_{0}}$ and the equality in (53) is due to that $U_{n}^{T}U_{n}=I_{s_{0}}.$

To summarize, we’ve shown

(54)
$$\sum_{ijk}{\left({\left({\hat{a}}_{i}^{\left(s\right)}\right)}^{T}{\hat{R}}_{k}^{\left(s\right)}{\hat{a}}_{j}^{\left(s\right)}-{\left(a_{i}^{*}\right)}^{T}R_{k}^{*}a_{j}^{*}\right)}^{2}\geq n^{2}{\left({\text{λ}}_{n}^{\left(s_{0}\right)}\right)}^{2}.$$

In the following, we provide a lower bound for ${\left({\text{λ}}_{n}^{\left(s_{0}\right)}\right)}^{2}.$ By definition, ${\left({\text{λ}}_{\text{n}}^{\left({\text{s}}_{\text{0}}\right)}\right)}^{\text{2}}$ is the *s*_0_-th largest eigenvalue of

$$\frac{1}{n^{2}}A_{0}B_{0}^{T}B_{0}A_{0}^{T}=\frac{1}{n^{2}}\sum_{k=1}^{K}A_{0}R_{k,0}^{T}A_{0}^{T}A_{0}R_{k,0}A_{0}^{T}.$$

We first provide an lower bound for $\lambda_{\min}(\sum_{k=1}^{K}R_{k,0}^{T}A_{0}^{T}A_{0}R_{k,0}/n).$ Let $\Sigma_{A}=A_{0}^{T}A_{0}/n.$ Consider the following eigenvalue decomposition:

$$\Sigma_{A}=U_{A}\Lambda_{A}U_{A}^{T},$$

for some orthogonal matrix ***U***_*A*_ and some diagonal matrix $\Lambda_{A}.$ Under Assumption (A2), the matrix

$$\Sigma_{A}-\bar{c}I_{s_{0}}$$

is positive semidefinite. As a result, the matrix

$$\sum_{k=1}^{K}R_{k,0}^{T}\left(\Sigma_{A}-\bar{c}I_{s_{0}}\right)R_{k,0}$$

is positive semidefinite. Therefore, we have

(55)
$${\text{λ}}_{\min}\left(\sum_{k=1}^{K}R_{k,0}^{T}\Sigma_{A}R_{k,0}\right)=\underset{a_{0}\in {\mathbb{R}}^{s_{0}},{\left\|a_{0}\right\|}_{2}=1}{\inf}a_{0}^{T}\left(\sum_{k=1}^{K}R_{k,0}^{T}\Sigma_{A}R_{k,0}\right)a_{0}$$

$$\begin{array}{l} =\underset{a_{0}\in {\mathbb{R}}^{s_{0}},{\left\|a_{0}\right\|}_{2}=1}{\inf}\left\{a_{0}^{T}\left(\sum_{k=1}^{K}R_{k,0}^{T}\left(\Sigma_{A}-\bar{c}I_{s_{0}}\right)R_{k,0}\right)a_{0}+\bar{c}a_{0}^{T}\left(\sum_{k=1}^{K}R_{k,0}^{T}R_{k,0}\right)a_{0}\right\}\\ \geq \bar{c}\underset{a_{0}\in {\mathbb{R}}^{s_{0}},{\left\|a_{0}\right\|}_{2}=1}{\inf}a_{0}^{T}\left(\sum_{k=1}^{K}R_{k,0}^{T}R_{k,0}\right)a_{0}=\bar{c}\lambda_{\min}\left(\sum_{k=1}^{K}R_{k,0}^{T}R_{k,0}\right)=\bar{c}\bar{K}. \end{array}$$

By the eigenvalue decomposition, we have

$$\sum_{k=1}^{K}R_{k,0}^{T}\Sigma_{A}R_{k,0}=U_{RA}^{T}\Lambda_{RA}U_{RA},$$

for some orthogonal matrix $\Lambda_{RA}\in {\mathbb{R}}^{s_{0}\times s_{0}}$ and some diagonal matrix $U_{RA}\in {\mathbb{R}}^{s_{0}\times s_{0}}$. It follows from (55) that all the diagonal elements in $\Lambda_{RA}$ are positive. Let $\Lambda_{RA}^{1/2}$ be the diagonal matrix such that $\Lambda_{RA}^{1/2}\Lambda_{RA}^{1/2}=\Lambda_{RA}.$ Apparently, the diagonal elements in $\Lambda_{RA}^{1/2}$ are nonzero. Notice that

$$\frac{1}{n^{2}}\sum_{k=1}^{K}A_{0}R_{k,0}^{T}A_{0}^{T}A_{0}R_{k,0}A_{0}^{T}=\frac{1}{n}A_{0}U_{RA}^{T}\Lambda_{RA}^{1/2}U_{RA}U_{RA}^{T}\Lambda_{RA}^{1/2}U_{RA}A_{0}^{T}.$$

The *s*_0_ largest eigenvalues in $\frac{1}{n^{2}}\sum_{k=1}^{K}A_{0}R_{k,0}^{T}A_{0}^{T}A_{0}R_{k,0}A_{0}^{T}$ corresponds to the smallest eigenvalue in

$$\frac{1}{n}U_{RA}^{T}\Lambda_{RA}^{1/2}U_{RA}A_{0}^{T}A_{0}U_{RA}^{T}\Lambda_{RA}^{1/2}U_{RA}.$$

Similar to (55), we can show that

$$\;{\text{λ}}_{\min}\left(\frac{1}{n}U_{RA}^{T}\Lambda_{RA}^{1/2}U_{RA}A_{0}^{T}A_{0}U_{RA}^{T}\Lambda_{RA}^{1/2}U_{RA}\right)\geq \bar{c}\lambda_{\min}\left(U_{RA}^{T}\Lambda_{RA}^{1/2}U_{RA}U_{RA}^{T}\Lambda_{RA}^{1/2}U_{RA}\right)\;=\bar{c}{\text{λ}}_{\min}\left(U_{RA}^{T}\Lambda_{RA}U_{RA}\right)\geq {\bar{c}}^{2}\lambda_{\min}\left(\sum_{k=1}^{K}R_{k,0}^{T}\Sigma_{A}R_{k,0}\right).$$

Combining this together with (55), we obtain that

$${\left({\text{λ}}_{n,k}^{\left(s_{0}\right)}\right)}^{2}\geq {\bar{c}}^{2}\bar{K}.$$

It follows from (54) that

$$\sum_{ijk}{\left({\left({\hat{a}}_{i}^{\left(s\right)}\right)}^{T}{\hat{R}}_{k}^{\left(s\right)}{\hat{a}}_{j}^{\left(s\right)}-{\left(a_{i}^{*}\right)}^{T}R_{k}^{*}a_{j}^{*}\right)}^{2}\geq n^{2}{\bar{c}}^{2}\bar{K}.$$

This completes the proof.

### C.4. Proof of Theorem 1

It suffices to show

(56)
$$\mathrm{Pr}\left(\text{IC}\left(s_{0}\right)>\underset{1\leq s<s_{0}}{\max}\text{IC}(s)\right)\to 1,$$

and

(57)
$$\mathrm{Pr}\left(\text{IC}\left(s_{0}\right)>\underset{s_{0}\leq s\leq s_{\max}}{\max}\text{IC}(s)\right)\to 1.$$

We first show (56). Combining Lemma 3 with (31), we obtain that

$$2l_{0}\left({\left\{a_{i}^{*}\right\}}_{i},{\left\{R_{k}^{*}\right\}}_{k}\right)-2l_{0}\left({\left\{{\hat{a}}_{i}^{\left(s\right)}\right\}}_{i},{\left\{{\hat{R}}_{k}^{\left(s\right)}\right\}}_{k}\right)\geq \frac{\sum_{ijk}{\left(\theta_{ijk}^{*}-{\hat{\theta }}_{ijk}^{\left(s\right)}\right)}^{2}}{4\exp\left(\omega_{a}^{2}\omega_{r}\right)}\geq \frac{n^{2}{\bar{c}}^{2}\bar{K}}{4\exp\left(\omega_{a}^{2}\omega_{r}\right)},$$

for any $s\in \left\{1,\ldots ,s_{0}-1\right\}.$ Combining this with (42), we have that

(58)
$$\;2l_{n}\left(Y;{\left\{a_{i}^{*}\right\}}_{i},{\left\{R_{k}^{*}\right\}}_{k}\right)-2l_{n}\left(Y;{\left\{{\hat{a}}_{i}^{\left(s\right)}\right\}}_{i},{\left\{{\hat{R}}_{k}^{\left(s\right)}\right\}}_{k}\right)\geq 2l_{0}\left({\left\{a_{i}^{*}\right\}}_{i},{\left\{R_{k}^{*}\right\}}_{k}\right)-2l_{0}\left({\left\{{\hat{a}}_{i}^{\left(s\right)}\right\}}_{i},{\left\{{\hat{R}}_{k}^{\left(s\right)}\right\}}_{k}\right)\;-2\left|l_{n}\left(Y;{\left\{a_{i}^{*}\right\}}_{i},{\left\{R_{k}^{*}\right\}}_{k}\right)-l_{0}\left({\left\{a_{i}^{*}\right\}}_{i},{\left\{R_{k}^{*}\right\}}_{k}\right)\right|-2\left|l_{n}\left(Y;{\left\{{\hat{a}}_{i}^{\left(s\right)}\right\}}_{i},{\left\{{\hat{R}}_{k}^{\left(s\right)}\right\}}_{k}\right)-l_{0}\left({\left\{{\hat{a}}_{i}^{\left(s\right)}\right\}}_{i},{\left\{{\hat{R}}_{k}^{\left(s\right)}\right\}}_{k}\right)\right|\;\geq \frac{{\bar{c}}^{2}n^{2}\bar{K}}{4\exp\left(\omega_{a}^{2}\omega_{r}\right)}-24\omega_{a}^{2}\omega_{r}n\sqrt{K\max(n,K)\log(nK)},$$

with probability tending to 1.

Under the given conditions, we have $K\sim n^{l_{0}}\;\text{for some}\;0\leq l_{0}\leq 1$. This together with the condition $n^{\left(1-l_{0}\right)/2}\bar{K}\gg \exp\left(2\omega_{a}^{2}\omega_{r}\right)\sqrt{\log n}$ yields

(59)
$$\omega_{a}^{2}\omega_{r}n\sqrt{K\max(n,K)\log(nK)}=O\left(\exp\left(\omega_{a}^{2}\omega_{r}\right)n^{3/2+l_{0}/2}\sqrt{\log n}\right)\ll \frac{n^{2}\bar{K}}{\exp\left(\omega_{a}^{2}\omega_{r}\right)}.$$

By (58), we have with probability tending to 1 that

(60)
$$2l_{n}\left(Y;{\left\{a_{i}^{*}\right\}}_{i},{\left\{R_{k}^{*}\right\}}_{k}\right)-2l_{n}\left(Y;{\left\{{\hat{a}}_{i}^{\left(s\right)}\right\}}_{i},{\left\{{\hat{R}}_{k}^{\left(s\right)}\right\}}_{k}\right)\geq \frac{{\bar{c}}^{2}n^{2}\bar{K}}{8\exp\left(\omega_{a}^{2}\omega_{r}\right)},$$

for all 1 ≤ *s* < *s*_0_. By definition, we have

$$l_{n}\left(Y;{\left\{{\hat{a}}_{i}^{\left(s_{0}\right)}\right\}}_{i},{\left\{{\hat{R}}_{k}^{\left(s_{0}\right)}\right\}}_{k}\right)\geq l_{n}\left(Y;{\left\{a_{i}^{*}\right\}}_{i},{\left\{R_{k}^{*}\right\}}_{k}\right).$$

This together with (60) gives that for all 1 ≥ *s* < *s*_0_,

(61)
$$2l_{n}\left(Y;{\left\{{\hat{a}}_{i}^{\left(s_{0}\right)}\right\}}_{i},{\left\{{\hat{R}}_{k}^{\left(s_{0}\right)}\right\}}_{k}\right)-2l_{n}\left(Y;{\left\{{\hat{a}}_{i}^{\left(s\right)}\right\}}_{i},{\left\{{\hat{R}}_{k}^{\left(s\right)}\right\}}_{k}\right)\geq \frac{{\bar{c}}^{2}n^{2}\bar{K}}{8\exp\left(\omega_{a}^{2}\omega_{r}\right)},$$

with probability tending to 1.

Under the given conditions, we have $\kappa (n,K)\ll n^{2}\bar{K}/\exp\left(\omega_{a}^{2}\omega_{r}\right).$ Under the event defined in (61), we have that

$$\text{IC}\left(s_{0}\right)-\text{IC}(s)=2l_{n}\left(Y;{\left\{{\hat{a}}_{i}^{\left(s_{0}\right)}\right\}}_{i},{\left\{{\hat{R}}_{k}^{\left(s_{0}\right)}\right\}}_{k}\right)-2l_{n}\left(Y;{\left\{{\hat{a}}_{i}^{\left(s\right)}\right\}}_{i},{\left\{{\hat{R}}_{k}^{\left(s\right)}\right\}}_{k}\right)-\left(s_{0}-s\right)\kappa (n,K)\;\;\geq \frac{{\bar{c}}^{2}n^{2}\bar{K}}{8\exp\left(\omega_{a}^{2}\omega_{r}\right)}-s_{0}\kappa (n,K)\gg 0,$$

since *s*_0_ is fixed. This proves (56).

Now we show (57). Similar to (37)-(42), we can show the following event occurs with probability tending to 1,

(62)
$$\underset{s\in \left\{s_{0},\ldots ,s_{\max}\right\}\;a_{1}^{\left(s\right)},\ldots ,a_{n}^{\left(s\right)}\in \Omega_{a},R_{1}^{\left(s\right)},\ldots ,R_{K}^{\left(s\right)}\in \Omega_{r}\;\frac{d\{{\left\{a_{i}^{\left(s\right)}\right\}}_{i},{\left\{R_{k}^{\left(s\right)}\right\}}_{k};{\left\{a_{i}^{*}\right\}}_{i},{\left\{R_{k}^{*}\right\}}_{k})}{s\exp\left(\omega_{a}^{2}\omega_{r}\right)}=O\left(\frac{r_{n,K}^{-1}}{n\sqrt{K}}\right)}{\sup}\frac{1}{s^{2}}\left|\sum_{ijk}\left(Y_{ijk}-\pi_{ijk}^{*}\right)\left\{\theta_{ijk}^{*}-{\left(a_{i}^{\left(s\right)}\right)}^{T}R_{k}^{\left(s\right)}a_{j}^{\left(s\right)}\right\}\right|=O\left(\frac{\exp\left(\omega_{a}^{2}\omega_{r}\right)}{r_{n,K}^{2}}\right).$$

By Lemma 2, we obtain with probability tending to 1,

$$\underset{s\in \left\{s_{0},\ldots ,s_{\max}\right\}}{\max}\frac{1}{s^{2}}\left|\sum_{ijk}\left(Y_{ijk}-\pi_{ijk}^{*}\right)\left\{\theta_{ijk}^{*}-{\left({\hat{a}}_{i}^{\left(s\right)}\right)}^{T}{\hat{R}}_{k}^{\left(s\right)}{\hat{a}}_{j}^{\left(s\right)}\right\}\right|=O\left(\frac{\exp\left(\omega_{a}^{2}\omega_{r}\right)}{r_{n,K}^{2}}\right),$$

and hence

(63)
$$\underset{s\in \left\{s_{0},\ldots ,s_{\max}\right\}}{\max}\frac{1}{s}\left|\sum_{ijk}\left(Y_{ijk}-\pi_{ijk}^{*}\right)\left\{\theta_{ijk}^{*}-{\left({\hat{a}}_{i}^{\left(s\right)}\right)}^{T}{\hat{R}}_{k}^{\left(s\right)}{\hat{a}}_{j}^{\left(s\right)}\right\}\right|=O\left(\frac{s_{\max}\exp\left(\omega_{a}^{2}\omega_{r}\right)}{r_{n,K}^{2}}\right).$$

Since $l_{0}\left({\left\{a_{i}^{*}\right\}}_{i},{\left\{R_{k}^{*}\right\}}_{k}\right)\geq l_{0}\left({\left\{a_{i}^{*}\right\}}_{i},{\left\{R_{k}^{*}\right\}}_{k}\right),$ under the event defined in (63), we have

$$\;2l_{n}\left(Y;{\left\{{\hat{a}}_{i}^{\left(s\right)}\right\}}_{i},{\left\{{\hat{R}}_{k}^{\left(s\right)}\right\}}_{k}\right)-2l_{n}\left(Y;{\left\{a_{i}^{*}\right\}}_{i},{\left\{R_{k}^{*}\right\}}_{k}\right)\leq 2l_{0}\left({\left\{{\hat{a}}_{i}^{\left(s\right)}\right\}}_{i},{\left\{{\hat{R}}_{k}^{\left(s\right)}\right\}}_{k}\right)-2l_{0}\left({\left\{a_{i}^{*}\right\}}_{i},{\left\{R_{k}^{*}\right\}}_{k}\right)\;-2\left|\sum_{ijk}\left(Y_{ijk}-\pi_{ijk}^{*}\right)\left\{\theta_{ijk}^{*}-{\left({\hat{a}}_{i}^{\left(s\right)}\right)}^{T}{\hat{R}}_{k}^{\left(s\right)}{\hat{a}}_{j}^{\left(s\right)}\right\}\right|=O\left(\frac{ss_{\max}\exp\left(\omega_{a}^{2}\omega_{r}\right)}{r_{n,K}^{2}}\right).$$

Notice that

$$l_{0}\left({\left\{{\hat{a}}_{i}^{\left(s\right)}\right\}}_{i},{\left\{{\hat{R}}_{k}^{\left(s\right)}\right\}}_{k}\right)\leq l_{0}\left({\left\{a_{i}^{*}\right\}}_{i},{\left\{R_{k}^{*}\right\}}_{k}\right),$$

we have with probability tending to 1 that

(64)
$$2l_{n}\left(Y;{\left\{{\hat{a}}_{i}^{\left(s\right)}\right\}}_{i},{\left\{{\hat{R}}_{k}^{\left(s\right)}\right\}}_{k}\right)-2l_{n}\left(Y;{\left\{{\hat{a}}_{i}^{\left(s_{0}\right)}\right\}}_{i},{\left\{{\hat{R}}_{k}^{\left(s_{0}\right)}\right\}}_{k}\right)\leq O\left(\frac{ss_{\max}\exp\left(\omega_{a}^{2}\omega_{r}\right)}{r_{n,K}^{2}}\right),$$

for all $s_{0}<s\leq s_{\text{max}}.$ Under the event defined in (64), we have

$$\text{IC}\left(s_{0}\right)-\text{IC}(s)\geq 2l_{n}\left(Y;{\left\{{\hat{a}}_{i}^{\left(s_{0}\right)}\right\}}_{i},{\left\{{\hat{R}}_{k}^{\left(s_{0}\right)}\right\}}_{k}\right)-2l_{n}\left(Y;{\left\{{\hat{a}}_{i}^{\left(s\right)}\right\}}_{i},{\left\{{\hat{R}}_{k}^{\left(s\right)}\right\}}_{k}\right)+\left(s-s_{0}\right)\kappa (n,K)\;\;\geq \left(s-s_{0}\right)\kappa (n,K)-O\left(ss_{\max}\exp\left(\omega_{a}^{2}\omega_{r}\right)r_{n,K}^{-2}\right).$$

Under the condition $\kappa (n,K)\gg s_{\max}\exp\left(\omega_{a}^{2}\omega_{r}\right)(n+K)(\log n+\log K),$ we have that

$$\mathrm{Pr}\left(\text{IC}\left(s_{0}\right)>\underset{s_{0}<s\leq s_{\max}}{\max}\mathrm{IC}(s)\right)\to 1,$$

This proves (57). The proof is hence completed.

### C.5. Proof of Corollary 1

Using similar arguments in Lemma 3, we can show for any permutation function *π* : {1,…,n}→{1,…,n},

(65)
$$\sum_{ijk}{\left({\left({\hat{a}}_{i,\pi }^{\left(s\right)}\right)}^{T}{\hat{R}}_{k,\pi }^{\left(s\right)}{\hat{a}}_{j,\pi }^{\left(s\right)}-{\left(a_{i,\pi }^{*}\right)}^{T}R_{k,\pi }^{*}a_{j,\pi }^{*}\right)}^{2}\geq n^{2}{\bar{c}}^{2}\bar{K}.$$

In addition, it follows from (41) and the condition $K=O\left(n^{l_{0}}\right)$ that

$$\mathrm{Pr}\left(\underset{\begin{array}{c} s\in \left\{1,\ldots ,s_{\max}\right\}\\ \pi :\{1,\ldots ,n\}\to \{1,\ldots ,n\} \end{array}}{\max}\frac{1}{s}\left|\sum_{ijk}\left(Y_{ijk}-\pi_{ijk}^{*}\right){\left({\hat{a}}_{i,\pi }^{\left(s\right)}\right)}^{T}{\hat{R}}_{k,\pi }^{\left(s\right)}{\hat{a}}_{j,\pi }^{\left(s\right)}\right|\leq C_{0}\omega_{a}^{2}\omega_{r}n^{3/2+l_{0}/2}\sqrt{\log n}\right)\to 1,$$

and

$$\mathrm{Pr}\left(\frac{1}{s_{0}}\left|\sum_{ijk}\left(Y_{ijk}-\pi_{ijk}^{*}\right){\left(a_{i}^{*}\right)}^{T}R_{k}^{*}a_{j}^{*}\right|\leq C_{0}\omega_{a}^{2}\omega_{r}n^{3/2+l_{0}/2}\sqrt{\log n}\right)\to 1,$$

for some constant *C*_0_ > 0. Hence, the following event occurs with probability tending to 1,

(66)
$$\max(\underset{s,\pi }{\max}\left|l_{0}\left({\left\{{\hat{a}}_{i,\pi }^{\left(s\right)}\right\}}_{i},{\left\{{\hat{R}}_{k,\pi }^{\left(s\right)}\right\}}_{k}\right)-l_{n}\left(Y;{\left\{{\hat{a}}_{i,\pi }^{\left(s\right)}\right\}}_{i},{\left\{{\hat{R}}_{k,\pi }^{\left(s\right)}\right\}}_{k}\right)\right|/s,$$

$$\left|l_{0}\left({\left\{a_{i}^{*}\right\}}_{i},{\left\{R_{k}^{*}\right\}}_{k}\right)-l_{n}\left(Y;{\left\{a_{i}^{*}\right\}}_{i},{\left\{R_{k}^{*}\right\}}_{k}\right)\right|/s_{0})\leq C_{0}\omega_{a}^{2}\omega_{r}n\sqrt{K\max(n,K)\log(nK)}.$$

Combining (65) with (31) yields

(67)
$$2l_{0}\left({\left\{a_{i}^{*}\right\}}_{i},{\left\{R_{k}^{*}\right\}}_{k}\right)-2\underset{\begin{array}{c} s\in \left\{1,\ldots ,s_{0}-1\right\}\\ \pi :\{1,\ldots ,n\}\to \{1,\ldots ,n\} \end{array}}{\max}l_{0}\left({\left\{{\hat{a}}_{i,\pi }^{\left(s\right)}\right\}}_{i},{\left\{{\hat{R}}_{k,\pi }^{\left(s\right)}\right\}}_{k}\right)\geq \frac{n^{2}{\bar{c}}^{2}\bar{K}}{4\exp\left(\omega_{a}^{2}\omega_{r}\right)}.$$

Under the given conditions, we have

$$\frac{n^{2}{\bar{c}}^{2}\bar{K}}{4\exp\left(\omega_{a}^{2}\omega_{r}\right)}\gg \omega_{a}^{2}\omega_{r}n\sqrt{K\max(n,K)\log(nK)}.$$

This together with (66) and (67) gives

$$2l_{n}\left(Y;{\left\{a_{i}^{*}\right\}}_{i},{\left\{R_{k}^{*}\right\}}_{k}\right)-2\underset{\begin{array}{c} s\in \{1,\ldots ,s_{0}-1\}\\ \pi :\{1,\ldots ,n\}\to \{1,\ldots ,n\} \end{array}}{\max}l_{n}\left(Y;{\left\{{\hat{a}}_{i,\pi }^{\left(s\right)}\right\}}_{i},{\left\{{\hat{R}}_{k,\pi }^{\left(s\right)}\right\}}_{k}\right)\geq \frac{n^{2}{\bar{c}}^{2}\bar{K}}{8\exp\left(\omega_{a}^{2}\omega_{r}\right)},$$

with probability tending to 1.

Under Condition (A4), we have $l_{n}\left(Y;{\left\{{\hat{a}}_{i,\pi }^{\left(s_{0}\right)}\right\}}_{i},{\left\{{\hat{R}}_{k,\pi }^{\left(s_{0}\right)}\right\}}_{k}\right)\geq l_{n}\left(Y;{\left\{a_{i,\pi }^{\left(s_{0}\right)*}\right\}}_{i},\left\{R_{k,\pi }^{\left(s_{0}\right)*}\right\}\right)=l_{n}\left(Y;{\left\{a_{i}^{*}\right\}}_{i},{\left\{R_{k}^{*}\right\}}_{k}\right).$ Therefore, the following event occurs with probability tending to 1,

$$\underset{\pi :\{1,\ldots ,n\}\to \{1,\ldots ,n\}}{\min}2\left(l_{n}\left(Y;{\left\{{\hat{a}}_{i,\pi }^{\left(s_{0}\right)}\right\}}_{i},{\left\{{\hat{R}}_{k,\pi }^{\left(s_{0}\right)}\right\}}_{k}\right)-\underset{s\in \left\{1,\ldots ,s_{0}-1\right\}}{\max}l_{n}\left(Y;{\left\{{\hat{a}}_{i,\pi }^{\left(s\right)}\right\}}_{i},{\left\{{\hat{R}}_{k,\pi }^{\left(s\right)}\right\}}_{k}\right)\right)\geq \frac{n^{2}{\bar{c}}^{2}\bar{K}}{\exp\left(\omega_{a}^{2}\omega_{r}\right)}.$$

Under the given conditions in Corollary 1, we obtain

(68)
$$\mathrm{Pr}\left(\underset{\pi :\{1,\ldots ,n\}\to \{1,\ldots ,n\}}{\cap }\left\{{\text{IC}}_{\pi }\left(s_{0}\right)>\underset{1\leq s\leq s_{0}-1}{\max}{\text{IC}}_{\pi }(s)\right\}\right)\to 1.$$

Similar to (46) and (49), we can show

$$\;\mathrm{Pr}\left(\underset{\pi :\{1,\ldots ,n\}\to \{1,\ldots ,n\}}{\cup }\left\{\left(\left\{{\hat{a}}_{i,\pi }^{\left(s\right)}\right\},{\hat{R}}_{k,\pi }^{\left(s\right)}\right)\in S_{m}^{\left(s\right)}\right\}\right)\;\leq \mathrm{Pr}\left(\underset{\left({\left\{a_{i}^{\left(s\right)}\right\}}_{i},{\left\{R_{k}^{\left(s\right)}\right\}}_{k}\right)\in S_{m}^{\left(s\right)}}{\sup}\left|\sum_{ijk}\left(Y_{ijk}-\pi_{ijk}^{*}\right)\left\{\theta_{ijk}^{*}-{\left(a_{i}^{\left(s\right)}\right)}^{T}R_{k}^{\left(s\right)}a_{j}^{\left(s\right)}\right\}\right|\geq \frac{{\left(m-1\right)}^{2}s^{2}\exp\left(\omega_{a}^{2}\omega_{r}\right)}{8r_{n,K}^{2}}\right)\;\leq 2\exp\left(-K_{0}m^{2}s^{2}r_{n,K}^{-2}\right),$$

for some constant *K*_0_ > 0. Using similar arguments in the proof of Lemma 2, this yields with probability tending to 1,

$$\underset{\begin{array}{c} s\in \left\{s_{0},\ldots ,s_{\max}\right\}\\ \pi :\{1,\ldots ,n\}\to \{1,\ldots ,n\} \end{array}}{\max}\frac{1}{s^{2}}d^{2}\left({\left\{{\hat{a}}_{i,\pi }^{\left(s\right)}\right\}}_{i},{\left\{{\hat{R}}_{k,\pi }^{\left(s\right)}\right\}}_{k};{\left\{a_{i}^{*}\right\}}_{i},{\left\{R_{k}^{*}\right\}}_{k}\right)\leq \frac{\exp\left(2\omega_{a}^{2}\omega_{r}\right)(n+K)(\log n+\log K)}{n^{2}K}.$$

This together with (62) gives

$$\underset{s\in \left\{s_{0},\ldots ,s_{\max}\right\}}{\max}\frac{1}{s}\left|\sum_{ijk}\left(Y_{ijk}-\pi_{ijk}^{*}\right)\left\{\theta_{ijk}^{*}-{\left({\hat{a}}_{i,\pi }^{\left(s\right)}\right)}^{T}{\hat{R}}_{k,\pi }^{\left(s\right)}{\hat{a}}_{j,\pi }^{\left(s\right)}\right\}\right|=O\left(\frac{s_{\max}\exp\left(\omega_{a}^{2}\omega_{r}\right)}{r_{n,K}^{2}}\right),$$

with probability tending to 1. Using similar arguments in the proof of Theorem 1, we can show

$$\mathrm{Pr}\left(\underset{\pi :\{1,\ldots ,n\}\to \{1,\ldots ,n\}}{\cap }\left\{{\text{IC}}_{\pi }\left(s_{0}\right)>\underset{s_{0}<s\leq s_{\max}}{\max}{\mathrm{IC}}_{\pi }(s)\right\}\right)\to 1.$$

This together with (68) yields (16). The proof is hence completed.

## Appendix D. Additional Simulation Results

We simulate the response ${\left\{Y_{ijk}\right\}}_{ijk}$ from the following model:

$$\mathrm{Pr}\left(Y_{ijk}=1|{\left\{a_{i}\right\}}_{i},{\left\{R_{k}\right\}}_{k}\right)=\frac{\exp\left(a_{i}^{T}R_{k}a_{j}\right)}{1+\exp\left(a_{i}^{T}R_{k}a_{j}\right)},\;a_{1},a_{2},\ldots ,a_{n}\overset{iid}{\sim }N\left(0,{\left\{0.5^{\left|i-j\right|}\right\}}_{i,j=1,\ldots ,s_{0}}\right),\;R_{1}=R_{2}=\cdots =R_{K}=\text{diag}(\underset{s_{0}}{\underset{︸}{1,-1,1,-1,\ldots ,1,-1}}).$$

We use the same six settings as described in Section 4.2. In each setting, we further consider three scenarios, by setting *s*_0_ = 2, 4 and 6. Reported in Table 3 and 4 are the percentage of selecting the true models (TP) and the average of $\hat{s}$ selected by IC_0_, IC_0.5_, IC_1_ and BIC over 100 replications.

It can be seen from Table 4 and 5 that our proposed information criteria are consistent. In contrast, BIC fails in all settings. In addition, in the last two settings, TPs of IC_0.5_ and IC_1_ are larger than IC_0_ for most of the cases. As commented before, these differences are due to the finite sample correction term *τ_α_*(*n, K*).
